# Complex Chromosomal Rearrangements Mediated by Break-Induced Replication Involve Structure-Selective Endonucleases

**DOI:** 10.1371/journal.pgen.1002979

**Published:** 2012-09-27

**Authors:** Benjamin Pardo, Andrés Aguilera

**Affiliations:** Centro Andaluz de Biología Molecular y Medicina Regenerativa CABIMER, Universidad de Sevilla, Sevilla, Spain; National Cancer Institute, United States of America

## Abstract

DNA double-strand break (DSB) repair occurring in repeated DNA sequences often leads to the generation of chromosomal rearrangements. Homologous recombination normally ensures a faithful repair of DSBs through a mechanism that transfers the genetic information of an intact donor template to the broken molecule. When only one DSB end shares homology to the donor template, conventional gene conversion fails to occur and repair can be channeled to a recombination-dependent replication pathway termed break-induced replication (BIR), which is prone to produce chromosome non-reciprocal translocations (NRTs), a classical feature of numerous human cancers. Using a newly designed substrate for the analysis of DSB–induced chromosomal translocations, we show that Mus81 and Yen1 structure-selective endonucleases (SSEs) promote BIR, thus causing NRTs. We propose that Mus81 and Yen1 are recruited at the strand invasion intermediate to allow the establishment of a replication fork, which is required to complete BIR. Replication template switching during BIR, a feature of this pathway, engenders complex chromosomal rearrangements when using repeated DNA sequences dispersed over the genome. We demonstrate here that Mus81 and Yen1, together with Slx4, also promote template switching during BIR. Altogether, our study provides evidence for a role of SSEs at multiple steps during BIR, thus participating in the destabilization of the genome by generating complex chromosomal rearrangements.

## Author Summary

Genome rearrangements consisting of non-reciprocal translocations (NRTs) seem to play an important role in carcinogenesis in humans. They are likely caused by intracellular mechanisms that are normally committed to repair breaks occurring in the DNA molecule. Failure of faithful repair of DNA double-strand breaks (DSBs) often leads to chromosomal rearrangements when repair occurs within repeated genomic regions. The break-induced replication (BIR) pathway of DSB repair is a major source of complex chromosomal rearrangements, the latter occurring when BIR involves template switching between dispersed repeated sequences. Given the deleterious consequences of such events for genomic stability, it is of great significance to understand the molecular bases of BIR. Here, we examined the role of different DNA nucleases in chromosomal rearrangements and uncovered the functional involvement of the structure-selective endonucleases (SSEs) subunits Mus81, Yen1, and Slx4 at different steps during BIR. Our work provides new clues to understand the origin of NRTs and the role of SSEs in their generation.

## Introduction

The maintenance of genome integrity is crucial to prevent cell death in all organisms. Chromosomal rearrangements such as reciprocal translocations, deletions, inversions and duplications threaten genomic stability and must be avoided to prevent cancer development and genomic disorders [1]. The occurrence of unfaithful repair of DNA double-strand breaks (DSBs) is widely admitted to be the main source of chromosomal rearrangements [2]–[5]. Nonhomologous End Joining (NHEJ) and Homologous recombination (HR) constitute the main pathways of DSB repair. While NHEJ seal the broken DNA ends by simple religation, HR uses sequence homology between the DSB ends and an intact template for repair and is typically considered as error free. Nevertheless, in a number of cases, such as when HR occurs between non-allelic DNA sequences or DNA repeated sequences or HR is used for the repair of DSB ends containing different levels of similarity, irreversible genomic changes can take place. Thus, when only one end of a DSB shares homology with other sequences in the genome, repair by HR can occur through a replication mechanism termed break-induced replication (BIR) that often gives rise to non-reciprocal translocations (NRTs) [6]–[9].

BIR requires HR canonical factors such as Rad52 and Rad51 to allow efficient strand invasion of the repair template and to form a Displacement-loop (D-loop) that can be extended by DNA synthesis from the invading 3′ DSB end [10], [11]. In the absence of another DSB end to capture the newly synthesized strand or to independently invade the homologous template, the strand invasion intermediate is thought to be converted into a DNA replication fork capable of replicating an entire chromosome arm until encountering a telomere, a centromere, or a converging replication fork [12]–[15]. A key factor in this process is Pol32, the non-essential subunit of DNA polymerase δ, which is dispensable for normal replication but essential for BIR [13]. Another feature of BIR is the unstable nature of its replication intermediates. It has been shown that DSB repair by BIR can occur through several rounds of strand invasion, synthesis, and dissociation from the invaded template [16]. Within dispersed repeated sequences, template switching during BIR can generate complex chromosomal rearrangements [16]–[21]. BIR reactions can also be aborted to end in half-crossovers [22], [23]. Half-crossovers cause NRTs, leaving the template that has been used for repair broken. They are similar to the NRTs observed in humans, which are involved in the cascade of genomic instability characteristic of human cancer cells [24].

Little is known about how BIR intermediates are processed to allow the establishment of a replication fork after strand invasion, and to cause template switching and half-crossovers. Eukaryotic cells have evolved a set of DNA structure-selective endonucleases (SSEs) that possess different substrate specificity for various DNA branched molecules during HR, such as D-loops, replication forks, flaps and Holliday junctions (HJs). Conversion of a D-loop into a replication fork during BIR may require the endonucleolytic resolution of a single Holliday junction [16], [25]. In budding yeast, Mus81-Mms4 and Yen1 are the only nuclear enzymes capable of cleaving intact HJs with high efficiency *in vitro* [26], [27]. *In vivo*, their functions seem to overlap during DSB repair [28]–[31]. Notably, Mus81-Mms4 is required for recombination-mediated DNA repair at replication forks [32]–[34] and has been shown to play a role in BIR intermediate processing [23]. Additionally, the Slx1–Slx4 nuclease complex may cleave perturbed replications forks [35]–[37]. Loss of Slx1–Slx4, as loss of Mus81-Mms4, increases the number of gross chromosomal rearrangements in yeast [38]. Slx4 also acts independently of the Slx1 catalytic subunit to interact with several other factors. For instance, Slx4 binds to the 3′ flap endonuclease complex Rad1–Rad10 to facilitate the removal of non-homologous tails during HR [39], [40]. Rad1–Rad10 complex, which functions in nucleotide excision repair (NER) as well as in DSB repair, has also been involved in the formation of translocations in yeast through a single-strand annealing (SSA) mechanism [41], [42]. Together, these SSEs form a complex network to ensure genome stability but their specific roles and their interactions at recombination structures remain unclear.

In this study, we used a new assay that generates chromosomal rearrangements after DSB repair using dispersed repeated sequences. Chromosomal rearrangements involved multiple rounds of template switching and some events ended in half-crossovers, generating NRTs. We investigated the role of SSEs in the processing of BIR intermediates combining mutations of Mus81, Rad1, Yen1, Slx1 and Slx4. Our results show that these SSEs act at multiple steps during BIR. First, we uncovered that Mus81 and Yen1 function to allow efficient BIR, thus causing translocations. In the absence of Mus81, Yen1 and Slx4, we observed that template switching during BIR decreased significantly. Altogether, our results led to new insights into the BIR mechanism and the functional role of SSEs in chromosomal rearrangements.

## Results

### A new assay for DSB–induced chromosomal rearrangements

**Figure 1:**
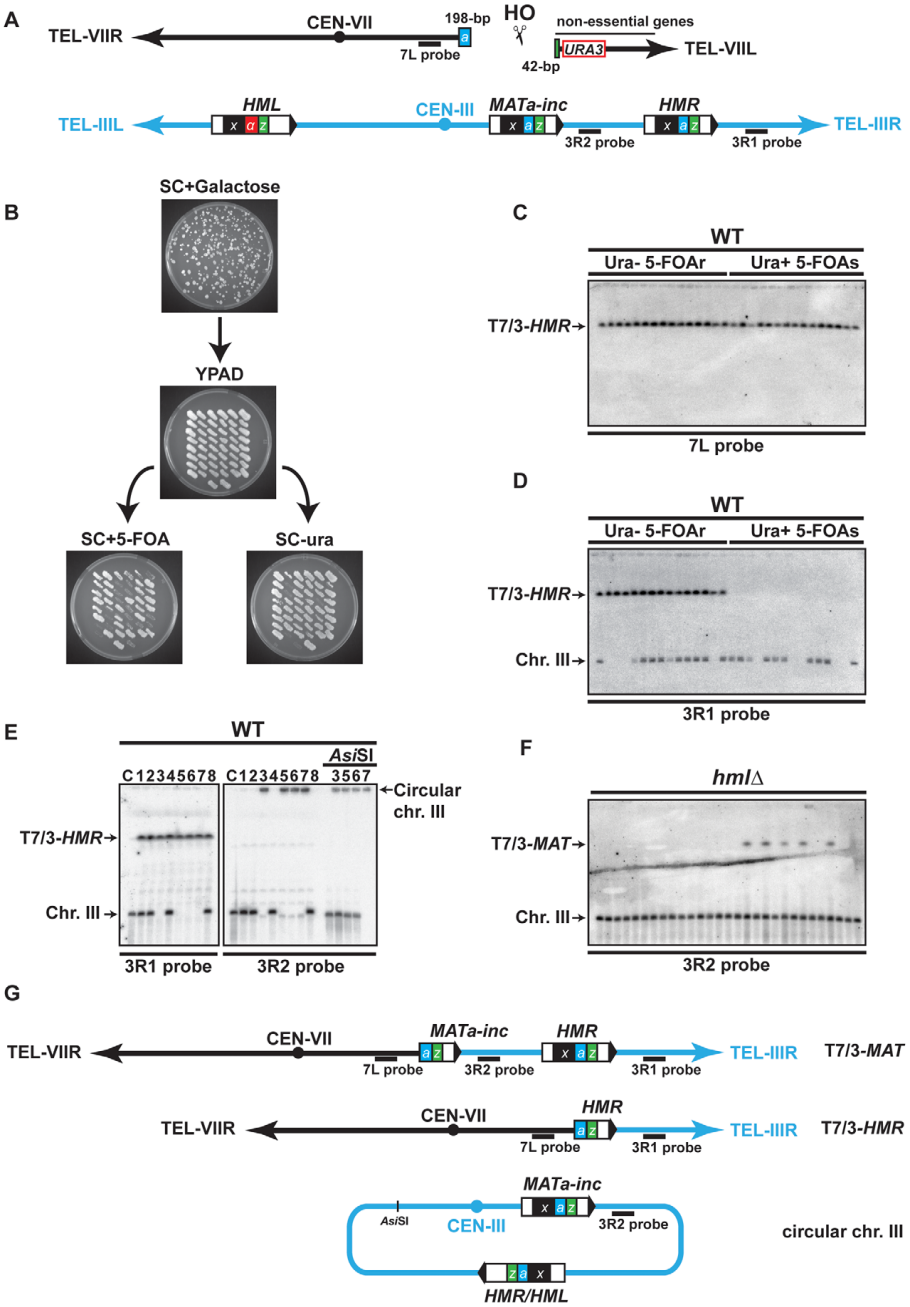
Description of the assay used in this study. (A) Schematic representation of chromosomes VII (black) and III (blue) in the haploid strain; X region (x, black box), Yα region (α, red box), Ya region (a, blue box) and Z1 region (z, green box) are indicated on chromosome III, as well as the Ya-Z1 fragment on chromosome VII. See text for more details. (B) Determination of translocation events. Cells were plated on solid synthetic medium containing galactose and survivor colonies were restreaked on rich YPAD medium containing glucose and subsequently replica-plated on SC+5-FOA and SC-ura. (C) (D) Translocations between chromosomes VII and III were detected by PFGE followed by Southern analysis using the 7L and the 3R1 probe (see panel A). Chromosomal DNA samples were extracted from previously selected Ura^−^ 5-FOAr survivor colonies. (E) Detection of circular chromosome III after PFGE and Southern analysis. Chromosomes III that were not hybridized with the 3R1 probe were detected in the gel wells using 3R2 probe (see panel A). The DNA samples corresponding to circular chromosome III were digested with *Asi*SI to linearize chromosome III. C, control strain without translocation. (F) PFGE followed by Southern analysis using the 3R2 probe of Ura^−^ 5-FOAr survivors in *hmlΔ* strain. Black arrows indicate the positions of the T7/3-*MAT* and the T7/3-*HMR* translocations and the circular and linear chromosomes III (Chr. III). PFGE, pulse-field gel electrophoresis. 5-FOAr and 5-FOAs refers to colonies either resistant or sensitive to 5-FOA. (G) Schematic representation of the predominant chromosomal rearrangements observed in this study.

We designed an experimental system using DNA repeated sequences dispersed over two yeast chromosomes for the analysis of chromosomal rearrangements induced by a single DSB. We took advantage of the presence of the *MAT*, *HMR* and *HML* loci on chromosome III, involved in mating-type switching. The *MAT* locus is composed of five regions called W, X, Y, Z1 and Z2 [43]. *MAT* **a** differs from *MATα* by the Y**a** and the Y*α* sequences, respectively. *MAT* **a** shares Y**a** with *HMR* and *MAT*α shares Y*α* with *HML*. Together, *MAT*, *HMR* and *HML* share two homologous regions flanking the Y sequences, termed X and Z1 (see Figure 1A). All three loci contain a cleavage site for the HO endonuclease at the junction between the Y and Z1 regions, but only the *MAT* locus is susceptible to being cleaved upon HO expression because of the active repression of *HMR* and *HML* loci [44]. The strains used in this study harbor a *MAT* **a** *-inc* mutation, a G to A substitution at position Z1–2, which impedes HO cleavage at *MAT* [45], [46]. The HO endonuclease gene under control of the *GAL1* inducible promoter was integrated at the *ADE3* locus and a 240-bp Y**a**-Z1 fragment containing a HO-cleavable site, along with the *URA3* marker, was inserted in the chromosome VII left arm at the *ADH4* locus (Figure 1A). Upon galactose addition to the culture medium, HO would cleave this Y**a**-Z1 fragment asymmetrically into a centromeric 198-bp fragment and a telomeric 42-bp fragment (Figure 1A).

To assay DSB repair in this system, we plated cells on a synthetic medium (SC) with either 2% glucose or 2% galactose to assay survival after HO expression and successive breakage of chromosome VII. The WT strain exhibited a survival frequency of 82% (Figure S1), demonstrating efficient DSB repair. We restreaked survivor colonies from galactose-containing plates on glucose medium to repress HO expression. Then, survivors were concomitantly replica-plated on media lacking uracil and on media containing 5-FOA, a drug that generates a toxic metabolite in Ura^+^ cells, to distinguish between the colonies that maintained or lost the *URA3* marker of chromosome VII (Figure 1B). We recollected Ura^−^ 5-FOA-resistant (20%), Ura^+^ 5-FOA-sensitive (12%), and Ura^+^ 5-FOA-resistant colonies (68%). The latter contained both Ura^+^ and Ura^−^ cells and may result from differential repair of two DSBs generated on sister chromatids during the S or G2 phase of the cell cycle. To analyze mixed Ura^+^ 5-FOA-resistant colonies, we first separated Ura^+^ cells from 5-FOA-resistant cells by restreaking colonies on media lacking uracil or containing 5-FOA. Further analyses of 5-FOA-resistant and Ura^+^ cells were performed by pulse-field gel electrophoresis (PFGE) to look for chromosomal translocations. Southern analysis using a probe specific to chromosome VII, proximal to the DSB site, and a probe specific to the region between *HMR* locus and chromosome III telomere (7L probe and 3R1 probe, respectively, Figure 1A) revealed the presence of non-reciprocal translocations (NRTs) between chromosome VII and chromosome III in all 5-FOA-resistant cells (Figure 1C, 1D, and Figure S2A, S2B). NRTs were rarely observed in Ura^+^ cells (Figure S2B). Given a survival rate of 82%, the frequency of translocants (frequency of 5-FOA-resistant survivors among the whole population that did not undergo DSB induction) was 44% in the WT strain. We mainly observed two types of NRTs, which contained chromosome III sequences starting from the *MAT* or *HMR* loci to the telomere fused to chromosome VII at the break site (termed T7/3-*MAT* and T7/3-*HMR* translocations for more clarity, Figure 1G).

Interestingly, some survivors lacked linear chromosome III at its expected size in the PFGE analysis (Figure 1D, 1E). When we used a probe specific to the region between *MAT* and *HMR* loci (3R2 probe, Figure 1A), chromosome III was detected in the well, consistent with a particular structure that did not allow it to enter the gel (Figure 1E). One possibility was that chromosome III became circular, as had been observed more than three decades ago [47], [48]. To test this, we digested chromosomal DNA with *Asi*SI, which cuts chromosome III at a unique inner location. Indeed, *Asi*SI digestion released chromosome III into the gel (Figure 1E). Circular chromosomes III have been shown to be the product of recombination between unrepressed *HMR* and *HML* loci, which share extensive homology in the X and Z1 regions (Figure 1A) [47]. We observed that the size of the *Asi*SI linearized chromosome was concordant with a chromosome III that would have lost all subtelomeric sequences located beyond the *HM* loci. To further demonstrate that circular chromosomes III occurred by recombination between *HMR* and *HML*, we assayed whether chromosome III circularization would be seen in *hmlΔ* cells. We observed that, in the absence of *HML* locus, all survivors contained a linear chromosome III at its expected size (Figure 1F).

Chromosome III circularization pushed us to investigate the occurrence of a DSB at *HMR*. *HMR* is naturally packed into heterochromatin to repress its expression and also to impede cleavage by HO [44]. We asked if *HMR* invasion would release its repression and cause HO cleavage. We first assayed *HMR* cleavage by Southern at several times after induction of chromosome VII cleavage by HO and did not observe any cuts in the WT (Figure S3C). We only detected *HMR* cleavage in a *hmlΔ matΔ* strain, in which *HMR* cleavage represented about a third of chromosome VII cleavage by HO (17% *versus* 46% after 3 h of induction; Figure S3B, S3C). Indeed *HMR* cleavage was dependent on cleavage at chromosome VII since no cuts were observed in the *hmlΔ matΔ* strain without HO cut site on chromosome VII (Figure S3D). We concluded that the induction of a single DSB by HO in chromosome VII permitted another less efficient DSB in chromosome III at the *HMR* locus as a secondary event. The efficiency of *HMR* cleavage is expected to be even less than 17% in the WT strain since the *MAT* **a** *-inc* locus is also available as a template for strand invasion in this strain. *HMR* cleavage likely induced chromosome III circularization, which we observed in 50% of translocants in the WT strain. Apart from T7/3-*MAT*, T7/3-*HMR* translocations and chromosome III circularization, we also observed rare types of rearrangements of chromosome VII and III whose nature has not been addressed in this study.

Together, these results demonstrate that complex chromosomal rearrangements are occurring at a high frequency in our experimental system, allowing us to investigate the molecular and genetic bases of these events.

### DSB repair involves template switching between *MAT*a*-inc*, *HMR*, and *HML*

**Figure 2:**
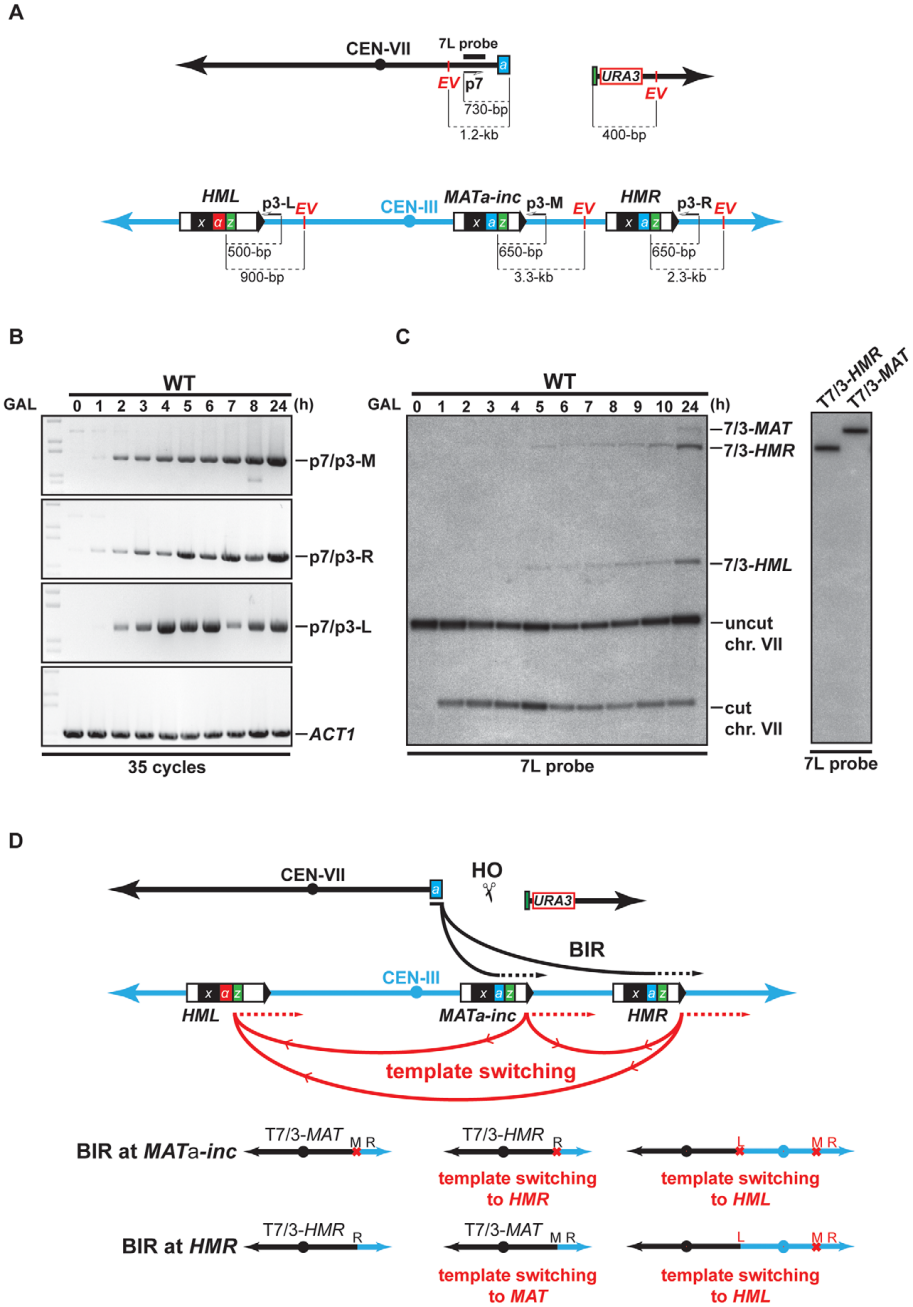
Template switching occurs between *MAT*a*-inc*, *HMR*, and *HML* loci. (A) Schematic representation of chromosomes VII and III showing the positions of the PCR primers, *Eco*RV sites (*E*V) and the 7L probe used for the analysis. Distances between the primers and *Eco*RV sites and the HO cut sites are indicated. (B) Appearance of BIR repair product, as monitored by PCR, in the WT strain. DNA samples used for PCR were extracted at intervals after HO induction with galactose. PCR reactions were performed with the p7 primer and either the p3-M, p3-R or p3-L primer (see panel A). A primer pair corresponding to *ACT1* locus on chromosome VI was used to control the amount of genomic DNA used for PCR at each time point. 35 cycles of PCR amplifications were performed. (C) Appearance of BIR repair products, as monitored by Southern analysis with the 7L probe, in the WT strain. DNA samples were extracted as for the PCR assay. Positions of the bands corresponding to 7/3-*MAT*, 7/3-*HMR* and 7/3-*HML* intermediates, and to the uncut and cut chromosome VII (chr. VII) are indicated. Two genomic DNA samples coming from survivor colonies containing T7/3-*MAT* and T7/3-*HMR* translocations were included in the experiment. GAL, galactose; h, hours. (D) Schematic representation of BIR events involving template switching likely occurring in our assay. Black arrows indicate the first invasion events while red arrows indicate the secondary template switching events. The different possible final outcomes are depicted. The red cross indicates the presence of *inc* non-cleavable sequences. M; *MAT*; R, *HMR*; L, *HML*. See text for more details.

We performed kinetic experiments to follow the recombination intermediates that gave rise to chromosomal rearrangements using our system. First, a PCR-based assay was used to monitor new DNA synthesis primed from the 3′ end of the invading strand of chromosome VII. Genomic DNA was extracted at different times after HO induction and PCR was made with one primer specific to chromosome VII (p7, Figure 2A), proximal to the DSB, and primers specific to each potential template used for recombination, *MAT* **a** *-inc* and *HMR* (p3-M and p3-R, Figure 2A). In WT cells, 35 cycles of PCR amplification permitted to detect products that most likely correspond to newly synthesized DNA fragments at both *MAT* and *HMR* loci 1 h after DSB induction (Figure 2B). Using more quantitative conditions, we detected the same products after 25 cycles of PCR amplification at 2 h of DSB induction (Figure S4). The amount of products increased over time. We then monitored the appearance of repair intermediates directly by Southern analysis of genomic DNA digested by *Eco*RV, using a probe specific to chromosome VII, proximal to the DSB site (7L probe). We detected recombination intermediates between chromosome VII and chromosome III at *HMR* (7/3-*HMR*, Figure 2C, left), which most likely correspond to newly synthesized DNA fragments 5 h after DSB induction. At *MAT*, we observed recombination intermediates 24 h after DSB induction (7/3-*MAT*, Figure 2C, left).

Southern analysis also detected an unexpected band that corresponded in size to chromosome VII fused to chromosome III sequences at the *HML* locus (7/3-*HML*, Figure 2C, left). We confirmed this assumption by re-probing the Southern membrane with a probe specific to *HML* (data not shown). The 7/3-*HML* band most likely corresponds to newly synthesized DNA fragments primed from chromosome VII DSB end at *HML* and appeared concomitantly with 7/3-*HMR* intermediates 5 h after DSB induction (Figure 2C). We monitored what was most likely new DNA synthesis primed from the 3′ DSB end of chromosome VII invading *HML* by PCR. We detected intermediates 2 h after DSB induction (p7/p3-L, Figure 2B, Figure S4). The DSB end of chromosome VII assayed by PCR and Southern only shares sequence homology with *MAT* **a** *-inc* and *HMR*. Hence, the signals detected at *HML* would be the consequence of a template switching from *MAT* **a** *-inc* or *HMR* to *HML* after duplication of the Z1 region that is common to the three loci. In a similar way, template switching could occur between *MAT* **a** *-inc* and *HMR*. 7/3-*HMR* and 7/3-*HML* intermediates detected by Southern appeared about 3–4 h after detection of priming of the 3′ invading DSB end by PCR (Figure 2B, 2C). This difference could be due to the difference of sensitivity between the two techniques. Alternatively, this could reflect a transition between the elongation of the 3′ invading DSB end, a step that is common to GC and BIR, and the establishment of an active replication fork required for BIR [11], [14]. Using translocants containing T7/3-*MAT* and T7/3-*HMR* translocations, we confirmed the size of the repair intermediates detected by Southern (Figure 2C, right). We did not recover any translocant containing translocations corresponding to repair intermediates detected at *HML*.

Together, these data show that one unique feature of our experimental system is that it allows the detection of events of template switching between *MAT* **a** *-inc* and *HML* and possibly between *MAT* **a** *-inc* and *HMR* during chromosomal rearrangement. These events of template switching likely participated to give rise to the formation of T7/3-*MAT* and T7/3-*HMR* translocations and dicentric chromosomes resulting from BIR completed from *HML* (Figure 2D). Because dicentric chromosomes are known to be unstable [49], this may be why we could not detect such type of chromosomal rearrangement. We noted that BIR initiation at *HMR* by the broken chromosome VII would restore an HO cleavable site that might not be properly silenced in the resulting translocations. In the latter case, HO cleavage would destabilize these translocations and their stabilization would require repair by gene conversion (GC) using the non-cleavable sequences at *MAT* **a** *-inc*.

### Mus81 and Yen1 promote chromosomal rearrangements

**Figure 3:**
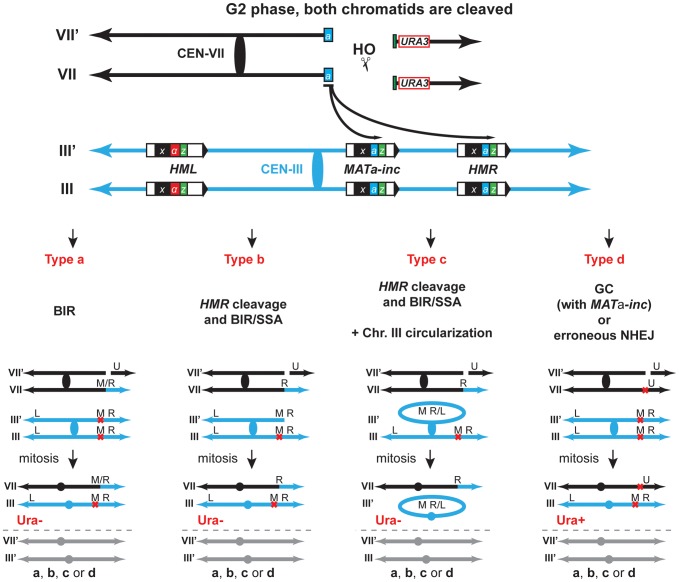
Various pathways give rise to chromosomal rearrangements. Schematic representation of the different types of repair giving rise to the chromosomal rearrangements scored in this study. Repair of DSBs in G2 can occur either by BIR, BIR/SSA following *HMR* cleavage with/without circularization of chromosome III, gene conversion (GC) or erroneous NHEJ. The repair types of only one DSB are depicted for simplification. The other DSBs are repaired via either one of the different types. VII, VII′ and III, III′ indicate chromosome VII and chromosome III sister-chromatids, respectively. U, *URA3*; M; *MAT*; R, *HMR*; L, *HML*; M/R, *MAT* or *HMR*. See text for more details.

Observed chromosomal rearrangements likely occurred by BIR through the invasion of the *MAT* **a** *-inc* and *HMR* loci. Homology of the centromeric 198-bp Y**a**-Z1 fragment present at one DSB end would be used to invade *MAT* **a** *-inc* or *HMR* loci at the Y**a** region and to duplicate chromosome III sequences until reaching its right telomere, generating T7/3-*MAT* and T7/3-*HMR* NRTs (type a, Figure 3). Alternatively, since we observed the cleavage of chromosome III as a consequence of *HMR* invasion, it is also possible that T7/3-*HMR* translocations occurred by BIR followed by single-strand annealing (SSA), co-segregating with an intact chromosome III (type b, Figure 3). Finally, cleaved chromosome III could circularize and co-segregate with the translocation (type c, Figure 3).

**Figure 4:**
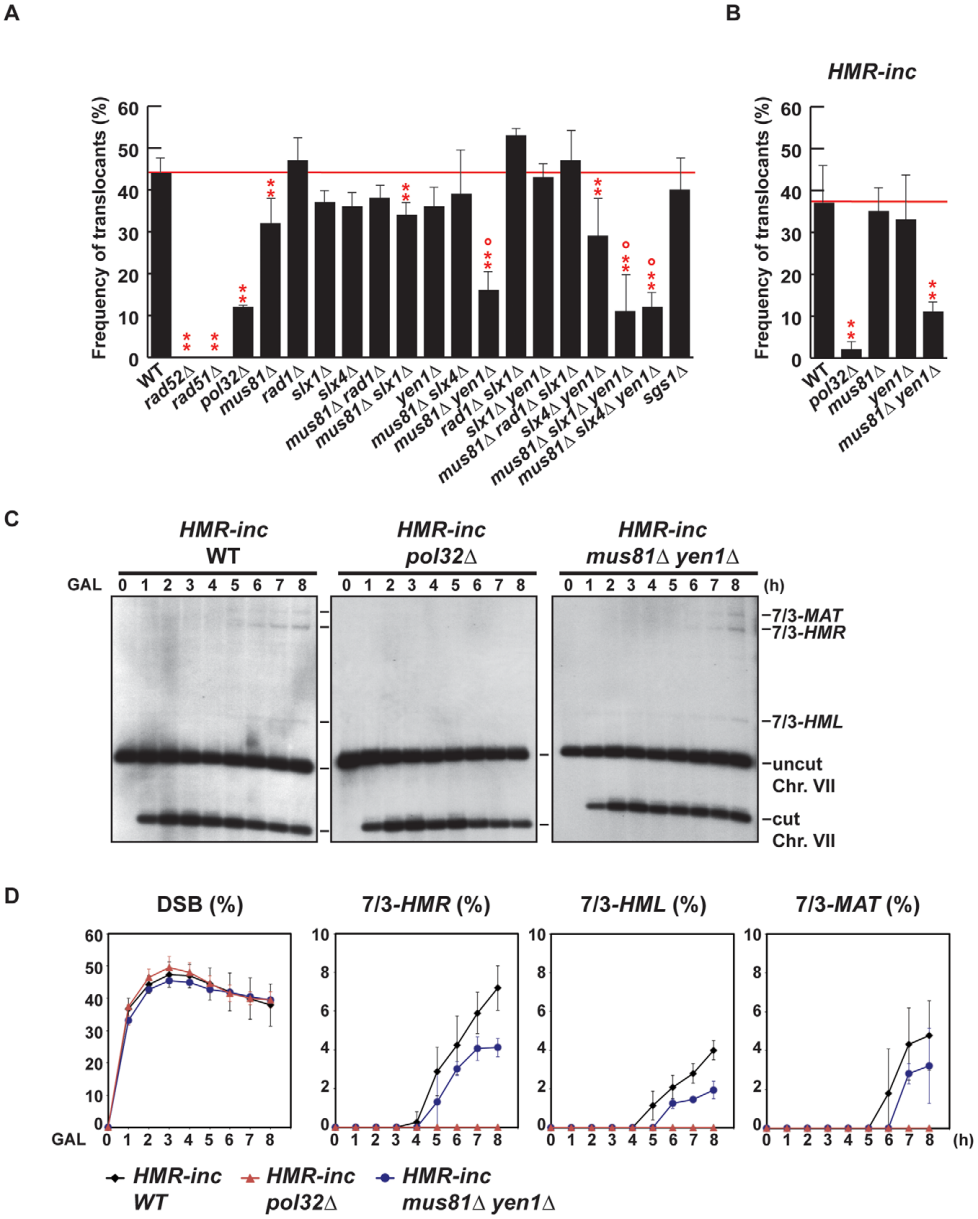
Genetic analysis of BIR intermediates processing in SSE mutants. (A) (B) Frequency of translocants of the different strains tested. See Material and Methods for details. ** and *, differences with the WT statistically significant (p<0.01 and p<0.05, respectively, χ^2^ with Yates' correction) °, statistically different from *mus81Δ* (p<0.01, χ^2^ with Yates' correction). Error bars represent standard deviations. (C) Appearance of BIR products as monitored by Southern analysis in *HMR-inc* WT, *HMR-inc pol32Δ* and *HMR-inc mus81Δ yen1Δ* strains. Experiments were performed as described in Figure 2C. A representative Southern analysis is shown for each genotype analyzed. Positions of the bands corresponding to 7/3-*MAT*, 7/3-*HMR* and 7/3-*HML* intermediates, and to the uncut and cut chromosome VII (Chr. VII) are indicated. GAL, galactose; h, hours. (D) Quantification of BIR product accumulation. Quantification results of chromosome VII cleavage (DSB), 7/3*-HMR*, 7/3*-HML* and 7/3*-MAT* BIR intermediates are shown as percentage. Mean values and standard deviations for 2–3 independent experiments are shown.

To confirm genetically the involvement of BIR in the generation of translocations using our experimental system, we assayed mutants of Rad52, Rad51 and Pol32 as representative key functions in BIR. Next, we asked whether DNA nucleases acting on branched structures such as D-loops, replication forks or HJs would have a role in the cascade of recombination events that led to translocations. We chose to study the genetic role of Mus81, Rad1, Yen1, Slx1 and Slx4 because of their known functions in recombination processes [25]. Because none of the genes coding for these SSEs are essential for viability, we assayed deletion mutants and combined mutants between them to assay redundancy of functions. Although Slx1 and Slx4 form a heterodimer complex, such as Mus81-Mms4 and Rad1–Rad10, we decided to study *slx1Δ* and *slx4Δ* mutations separately because Slx4 seems to have additional roles in DSB repair apart from regulating the Slx1 nuclease activity [50], [51]. We also included the DNA helicase Sgs1 because of its known role in HJ dissolution (Figure 4A).

For each mutant assayed, we recovered survivors after HO endonuclease induction and selected colonies that contained translocations between chromosome VII and chromosome III, as described above (5-FOA-resistant colonies). We did not recover any translocants in *rad52Δ* and *rad51Δ* mutants, in which strand invasion fails to occur (Figure 4A). *POL32* deletion reduced 4-fold the frequency of translocations in comparison with the WT (χ^2^, p<0.01), arguing in favor of the involvement of the Pol32-dependent BIR pathway in the generation of the translocations analyzed in this study. We did not observe any increase of translocations in *sgs1Δ* cells, even though *SGS1* gene had been identified as a suppressor of translocations involving template switching events [19]. Among the nuclease single mutants, only *mus81Δ* showed a slight but significant decrease in the frequency of translocants (χ^2^, p<0.01) when compared to the WT. In contrast, we observed significant decreases in the frequency of translocants between the WT, *mus81Δ rad1Δ* and *mus81Δ rad1Δ slx1Δ* mutants, but these effects were found to be epistatic with *mus81Δ* (Figure 4A; χ^2^, p<0.01). On the contrary, we observed further significant decreases (χ^2^, p<0.01) in *mus81Δ yen1Δ* mutants, of about 3.5-fold and 2.5-fold compared to the WT and *mus81Δ*, respectively (Figure 4A). We conclude that Mus81 and Yen1 are both required for translocations in our assay.

Since it existed the possibility that the translocations were also produced by BIR/SSA, we assayed translocation formation in an *HMR-inc* strain, in which *HMR* locus would not be susceptible to HO cleavage and translocations would only be produced by BIR. We observed that survival dropped from 82% in the original *HMR* WT strain to 52% in the *HMR-inc* WT strain, although the frequency of translocants remained around 40% (Figure 4B and Figure S1). In the control *HMR-inc* background, *POL32* deletion reduced 18.5-fold the frequency of translocations in comparison with the WT (Figure 4B; χ^2^, p<0.01). This result demonstrates a clear dependency of translocations on the Pol32-dependent BIR pathway in this background. We observed a significant decrease of 3.4-fold in *HMR-inc mus81Δ yen1Δ* mutants compared to the *HMR-inc* WT but no decrease in the *HMR-inc mus81Δ* and *HMR-inc yen1Δ* single mutants (Figure 3B; χ^2^, p<0.01). These results indicate that Mus81 and Yen1 have overlapping functions in BIR. To confirm this, we performed time-course experiments to monitor the kinetics of appearance of BIR intermediates in these mutants (Figure 4C). Kinetics of DSB formation in the mutants was similar to the WT, allowing us to directly compare the accumulation of BIR intermediates at each time point (Figure 4D). In *HMR-inc* WT cells, 7/3-*HMR* and 7/3-*HML* intermediates appeared 4 h and 5 h after DSB induction, respectively (Figure 4C, 4D). 7/3-*MAT* intermediates were detected 6 h after DSB induction (Figure 4C, 4D), showing that their delayed appearance in the original *HMR* WT strain was partly due to *HMR* cleavage. No BIR intermediate could be detected in *HMR-inc pol32Δ* cells (Figure 4C, 4D). In *HMR-inc mus81Δ yen1Δ* cells, a decrease of BIR intermediates was reproducibly observed at all time points (Figure 4C, 4D). We concluded that Mus81 and Yen1 are both required for promoting efficient Pol32-dependent BIR.

### Template switching is affected in structure-selective endonuclease mutants

**Figure 5:**
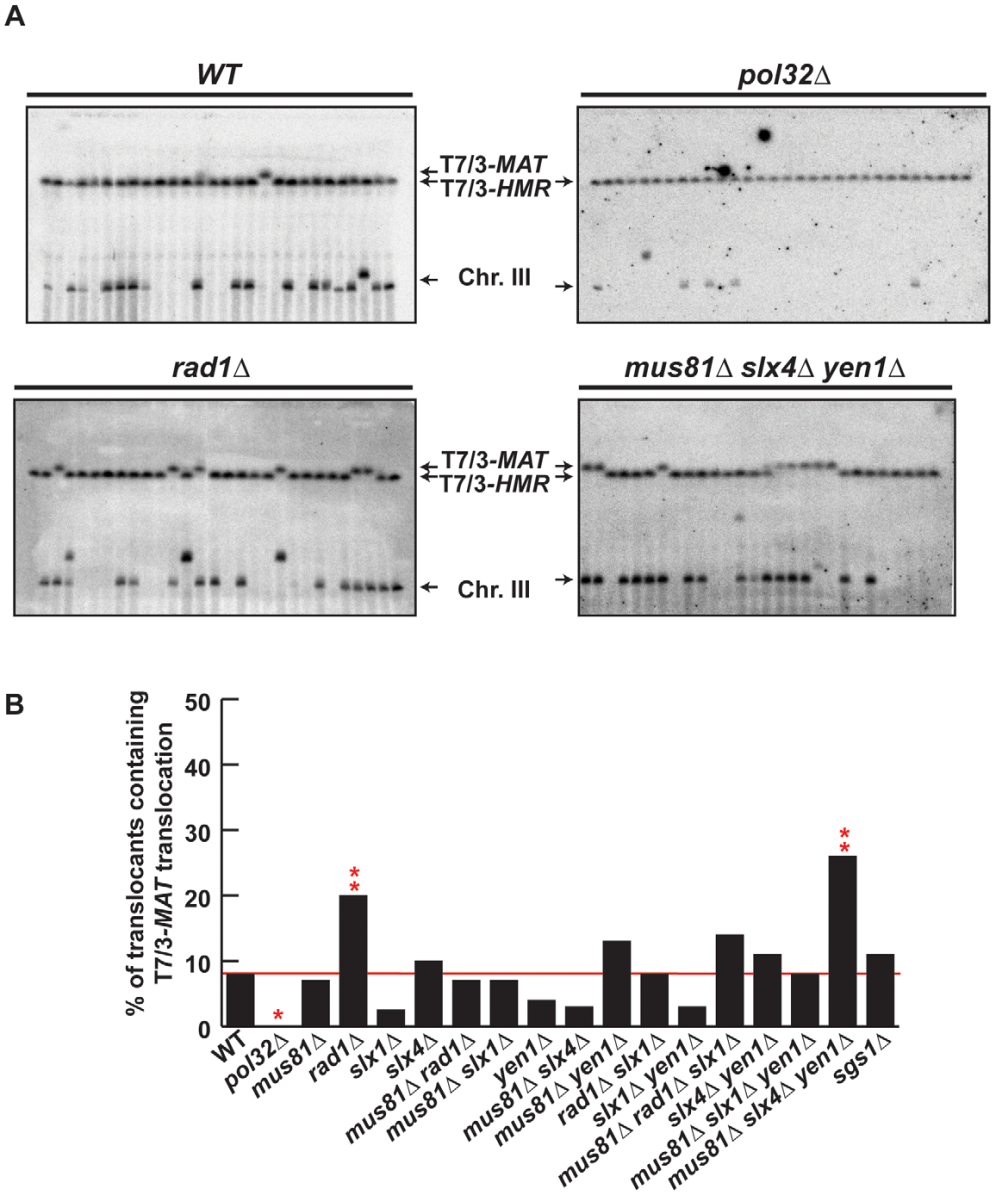
PFGE analysis of T7/3 translocations in SSE mutants. (A) PFGE followed by Southern analysis using the 3R1 probe (See Figure 1G) of Ura^−^ 5-FOAr survivors in WT, *pol32Δ*, *rad1Δ* and *mus81Δ slx4Δ yen1Δ* strains. Black arrows indicate the positions of the T7/3-*MAT* and T7/3-*HMR* translocations and of the linear chromosome III (Chr. III). PFGE, pulse-field gel electrophoresis. (B) Graphical plotting of percent of translocants containing T7/3-*MAT* translocations of each strain tested. ** and *, differences with the WT statistically significant (p<0.01 and p<0.05, respectively, χ^2^ with Yates' correction). See Table S1 for complete statistical analysis.

**Figure 6:**
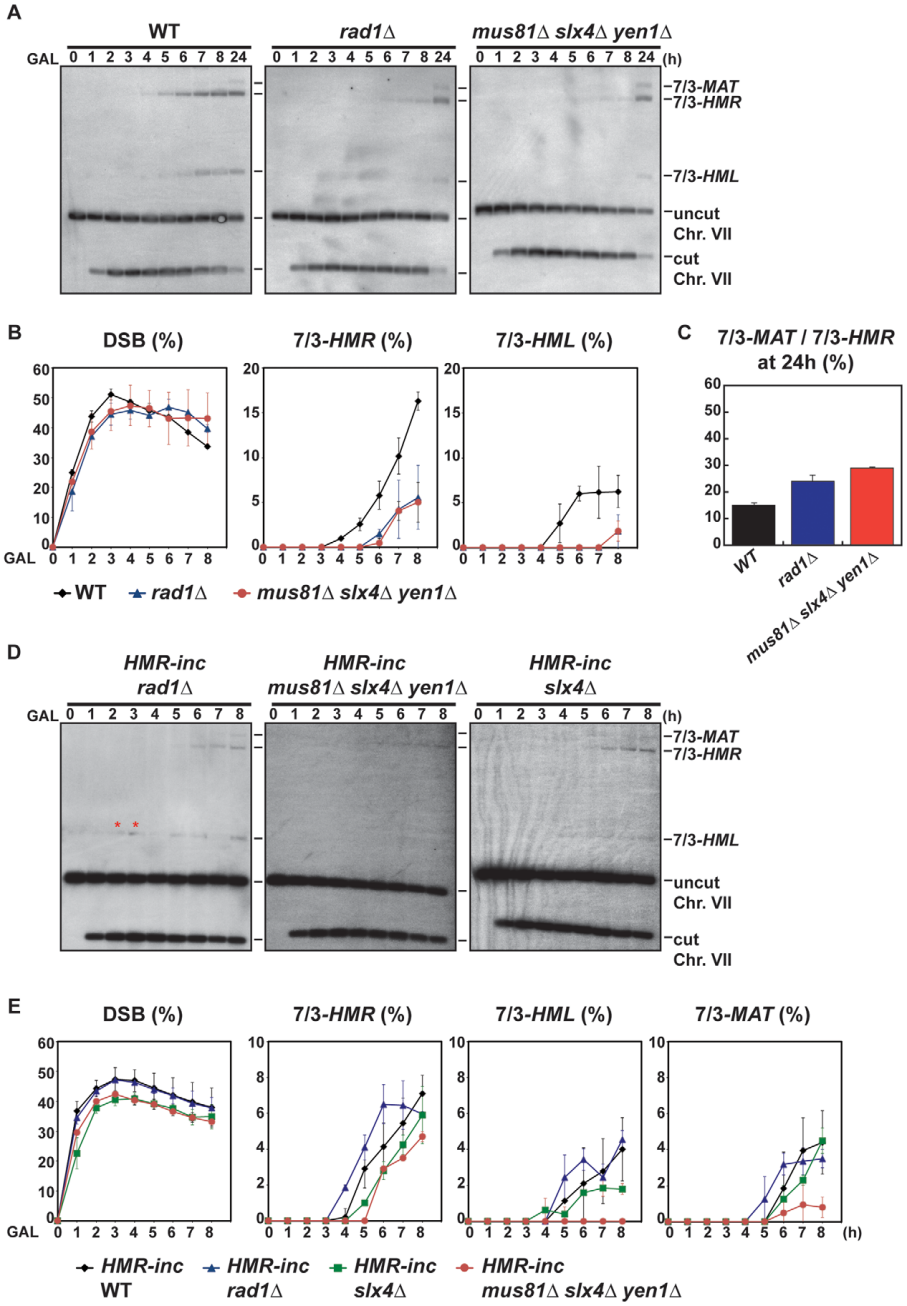
Template switching is affected in *mus81Δ slx4Δ yen1Δ* but not in *rad1Δ* mutants. (A) Appearance of BIR repair products, as monitored by Southern analysis, in WT, *rad1Δ* and *mus81Δ slx4Δ yen1Δ* strains. Experiments were performed as described in Figure 2C. (B) Quantification of BIR product accumulation. Quantification results for chromosome VII cleavage (DSB), 7/3*-HMR* and 7/3*-HML* BIR intermediate accumulation are shown in percent. (C) Quantification of 7/3*-MAT*/7/3*-HMR* BIR intermediate ratios 24 h after HO induction. Mean values and standard deviations for 2–3 independent experiments are shown. (D) Appearance of BIR repair products, as monitored by Southern analysis, in *HMR-inc rad1Δ*, *HMR-inc mus81Δ slx4Δ yen1Δ* and *HMR-inc slx4Δ* strains. (E) Quantification of BIR product accumulation. Quantification results for chromosome VII cleavage (DSB), 7/3*-HMR*, 7/3*-HML* and 7/3*-MAT* BIR intermediate accumulation are shown in percent. Mean values and standard deviations for 2–3 independent experiments are shown. A representative Southern analysis is shown for each genotype analyzed. Positions of the bands corresponding to 7/3-*MAT*, 7/3-*HMR* and 7/3-*HML* intermediates, and to the uncut and cut chromosome VII (Chr. VII) are indicated. Bands marked with a red asterisk likely result from partial digestion. GAL, galactose; h, hours.

We confirmed by PFGE that all 5-FOA-resistant survivors analyzed in mutant backgrounds contained chromosome translocations between chromosome VII and chromosome III (Figure 5A and Figures S5, S6). Notably, a very low amount of T7/3-*MAT* translocations (8%) were recovered in WT cells (Figure 5B). In *pol32Δ* cells, no T7/3-*MAT* translocation was recovered (n = 59, χ^2^, p<0.05), showing that this type of translocation has a complete dependency on Pol32 (Figure 5A, 5B). Among the nuclease mutants, *rad1Δ* showed a significant increase of T7/3-*MAT* translocations, up to 20% of total translocations (2.5-fold, n = 59, χ^2^, p<0.01), which was not observed in double mutants with *mus81Δ* and *slx1Δ* (Figure 5A, 5B). The concomitant absence of Mus81, Slx4 and Yen1 engendered an even higher increase (3.1-fold) of T7/3-*MAT* translocations that reached up to 25% of total translocations (n = 58, χ^2^, p<0.01) (Figure 5A, 5B). In kinetic experiments, both 7/3-*HMR* and 7/3-*HML* intermediates accumulated in the *rad1Δ* and *mus81Δ slx4Δ yen1Δ* mutants with kinetics clearly delayed (2–3 h) respect to the WT, demonstrating a defect of repair in these mutants (Figure 6A, 6B). At 24 h, signals for 7/3-*HMR*, 7/3-*HML* and 7/3-*MAT* that likely correspond to final repair products were detected in all strains. Notably, we observed a clear increase in the 7/3-*MAT*/7/3-*MAT* ratio, up to 24% and 29% in *rad1Δ* and *mus81Δ slx4Δ yen1Δ*, respectively, compared to the 15% seen in the WT (Figure 6C). This observation correlated with the increase of T7/3-*MAT* translocations previously observed in these mutants (Figure 5B).

The defects observed in *rad1Δ* and *mus81Δ slx4Δ yen1Δ* mutants could be explained by the known functions of Rad1 and Slx4 in SSA [50], which would be required for T7/3*-HMR* translocation formation by BIR/SSA. Indeed, in the control *HMR-inc* WT strain, the T7/3-*MAT* translocations represented about 55% of the translocations observed in 5-FOA-resistant survivors (Figure S7B). These results confirmed that the cleavage of the *HMR* locus upon its invasion in the *HMR* WT strain facilitated the formation of T7/3-*HMR* translocations as opposed to T7/3-*MAT* translocations. Additionally, possible cleavage of *HMR* upon the passage of the BIR fork initiated at *MAT* **a** *-inc* impaired the formation of T7/3-*MAT* translocations. Nevertheless, *slx4Δ* single mutants did not show any increase of T7/3-*MAT* translocations and *mus81Δ slx4Δ yen1Δ* mutants showed a higher increase of T7/3-*MAT* translocations than *rad1Δ* mutants (Figure 5B). We hypothesized that this was due to a processing defect of BIR intermediates formed at *MAT* **a** *-inc* that impeded subsequent template switching to *HMR* and *HML*. To explore this possibility, we analyzed the kinetics of appearance of BIR intermediates in *mus81Δ slx4Δ yen1Δ* mutants, as well as in the *rad1Δ* and *slx4Δ* single mutants, in the *HMR-inc* background (Figure 6D, 6E). In *HMR-inc* strains, BIR/SSA does not occur and the accumulation of 7/3-*HML* intermediates serves as an indicator of the efficiency of template switching during BIR. In contrast to the *HMR-inc rad1Δ* and *HMR-inc slx4Δ* mutants, which did not show any significant difference compared to the *HMR-inc* WT strain, *7/3-HML* intermediates were reproducibly not detected at all time points in *HMR-inc mus81Δ slx4Δ yen1Δ* mutants. Accumulation of 7/3-*HMR* and 7/3-*MAT* intermediates was also significantly lower in the latter strain, probably reflecting the BIR defect previously observed in *HMR-inc mus81Δ yen1Δ* cells.

Altogether, these data indicate that DSB repair was altered in cells lacking Rad1, defective in BIR/SSA, and in cells lacking all three SSE factors Mus81, Slx4 and Yen1 that show a defect of template switching during BIR.

### Chromosome III circularization and crossover activity

**Figure 7:**
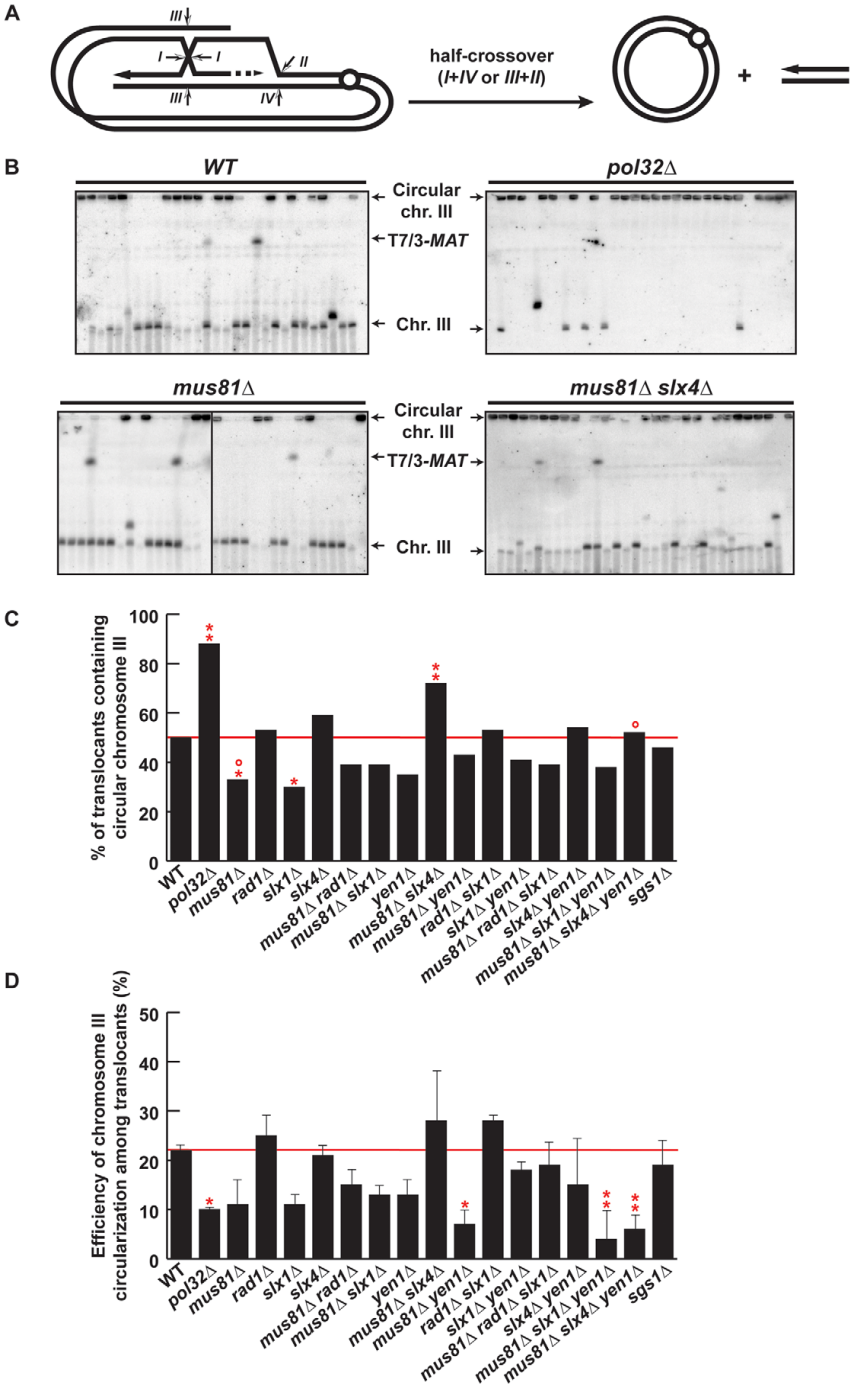
PFGE analysis of chromosome III circularization in SSE mutants. (A) Schematic representation of circularization of chromosome III by BIR plus half-crossover. Arrows indicate different ways of intermediate cleavage. (B) PFGE followed by Southern analysis using the 3R2 probe (See Figure 1G) of Ura^−^ 5-FOAr survivors in WT, *pol32Δ*, *mus81Δ*, *slx4Δ*, *mus81Δ slx4Δ* and *mus81Δ slx4Δ yen1Δ* strains. Black arrows indicate the positions of T7/3-*MAT* translocations, and the circular and linear chromosomes III (Chr. III). PFGE, pulse-field gel electrophoresis. (C) Graphical plotting of percent of translocants containing circular chromosome III for each strain tested. (D) Frequency of chromosome III circularization among translocants. ** and *, differences with the WT statistically significant (p<0.01 and p<0.05, respectively, χ^2^ with Yates' correction) **°**, differences with *mus81Δ slx4Δ* statistically significant (p<0.01, χ^2^ with Yates' correction). Error bars represent standard deviations. See Table S1 for complete statistical analysis.

Among the chromosomal rearrangements generated in our strains, circularization of chromosome III occurred at a high frequency. No chromosome III circularization was observed in the control *HMR-inc* WT strain (Figure S7B), demonstrating that *HMR* cleavage induced this secondary recombination event (type c, Figure 3). *HMR* cleavage and translocations via BIR/SSA would leave chromosome III with a one-ended DSB. Thus, circularization of chromosome III is thought to occur by a recombination event that ended in a half-crossover, characterized by a reciprocal exchange between *HMR* and *HML* that caused the loss of the chromosome III left telomere and the formation of a chromosome circle (Figure 7A). Circularization of chromosome III is not mandatory for survival of BIR/SSA-mediated translocants since the translocation can co-segregate with the uninvolved chromatid of chromosome III (type b, Figure 3). Therefore, we took advantage of chromosome III circularization events to investigate the ability of SSE mutant cells to produce half-crossovers.

PFGE analyses allowed us to detect circular chromosome III in all strains as signals appearing in the wells (Figure 7B and Figures S8, S9). In addition to the well signal, we also detected in some strains a faint signal for the truncated linear chromosome III. This faint signal corresponds in size to the circular chromosome III cleaved by *Asi*SI (Figure 1E). Since we did not find any linkage between the presence of this signal and a particular genetic background, we conclude that this is likely due to breakage occurring during DNA extraction or PFGE. To get further insight into the mechanism that gave rise to circular chromosomes, we have evaluated the percent of translocants that contained circular *versus* linear chromosomes III for each genotype (Figure 7C). Whereas about 50% of WT translocants contained a circular chromosome III, this percent increased significantly to 88% (n = 59, χ^2^, p<0.01) in *pol32Δ* mutants (Figure 7B, 7C). This result is concordant with previous observations showing that *pol32Δ* defects led to D-loop processing during BIR that generated half-crossovers [22], [23]. Among the SSE mutants tested, the proportion of translocants containing circular chromosomes III decreased significantly to 33% and 30% in *mus81Δ* and *slx1Δ* mutants, respectively (χ^2^, p<0.05) (Figure 7B, 7C). In contrast to this observation, the percentage of translocants containing circular chromosomes III increased significantly up to 72% in *mus81Δ slx4Δ* double mutants (χ^2^, p<0.01) (Figure 7B, 7C). These results suggest that Mus81 and Slx4 have different roles regarding crossover formation. Additionally, we observed that *yen1Δ* mutation suppressed the increase detected in *mus81 slx4Δ* mutants, suggesting that Yen1 action may be possible only in the absence of Slx4. We conclude that Slx4, which has been described as acting as a platform with different nuclease complexes [52], might regulate Mus81 and Yen1 accessibility to recombination intermediates or their nuclease activity to generate crossovers.

We also analyzed chromosome III circularization in Ura^+^ survivors, which did not contain translocations and likely performed DSB repair by GC or NHEJ (Figure S10). Analogously to what we observed in the Ura^−^ translocants, 36% of WT Ura^+^ survivors contained a circular chromosome III and this percentage increased significantly to 90% in *pol32Δ* mutants (Figure S10E). This suggests that chromosome III circularization does not depend on the translocation event and happens similarly in all survivors whether Ura^+^ or Ura^−^. Finally, we calculated the efficiency of chromosome III circularization for each genotype (Figure 7D). Pol32 appears to be required for efficient chromosome III circularization since the calculated efficiency went down significantly from 22% in WT to 10% in *pol32Δ* translocants (χ^2^, p<0.05) (Figure 7D). This probably explains the low survival of *pol32Δ* mutants to the DSB induction (Figure S1). Similarly, we observed a significant decrease in the efficiency of chromosome III circularization in *mus81Δ yen1Δ* mutants (Figure 7D). This is consistent with the conclusion that Mus81 and Yen1 play an important role in the formation of the majority of circular chromosomes III.

## Discussion

We developed an assay to study the molecular mechanisms that lead to complex chromosomal rearrangements upon induction of a DSB. DSB repair occurred by recombination between repeated DNA sequences dispersed over two different chromosomes and generated translocations and circularization of chromosomes at a high frequency. Our data indicate that chromosomal rearrangements occurred primarily by BIR, a HR sub-pathway that involves the invasion of an intact homologous DNA duplex by only one end of the DSB and subsequent replication primed from the invading strand. We have shown with this assay that the structure-selective endonuclease (SSE) factors Mus81, Yen1, and Slx4 may process recombination intermediates at different steps during BIR and cause template switching and half-crossovers.

In our assay, induction of the HO endonuclease produced a DSB on chromosome VII. Translocants can only be detected if both chromatids were broken in G2 phase (Figure 3), otherwise the DSB would be preferentially repaired by sister-chromatid recombination [53], [54], restoring the HO cut site. DSB ends on chromosome VII display homology to *MAT* and *HMR* loci that is mainly restricted to one end (198-bp out of 240-bp). As previously described, this feature favors DSB repair by BIR against a conventional gene conversion mechanism [11]. The centromeric 198-bp DSB end would invade *MAT* or *HMR* at the Y**a** region and prime BIR synthesis to produce the observed translocations, which contained chromosome III sequences from *MAT* or *HMR* to the telomere fused to chromosome VII at the break site, termed T7/3-*MAT* and T7/3-*HMR* translocations. Consequently, the loss of the chromosome VII arm, distal to the DSB and containing the *URA3* marker and non-essential genes, would lead to the 5-FOA-resistant (Ura^−^) phenotype of the translocants (type a, Figure 3). Eventually, the *URA3* marker could be used to convert the endogenous *ura3-1* allele on chromosome V and lead to the rarely observed Ura^+^ cells containing translocations (Figure S2B). Alternatively, secondary cleavage of *HMR* upon its invasion would promote repair by BIR/SSA and the production of the same types of translocations (type b, Figure 3). *HMR* cleavage would stimulate the circularization of chromosome III, which could co-segregate with the translocation (type c, Figure 3). Repair by BIR/SSA and chromosome III circularization do not happen in the *HMR-inc* background. The latter observation discards the possibility that the T7/3 translocations were the products of half-crossovers, with the translocation co-segregating with the uninvolved chromatid of chromosome III (analogous to type b, Figure 3), since breakage of chromosome III would stimulate its circularization. Lastly, DSB repair by GC with *inc* non-cleavable sequences or by erroneous NHEJ could seal the break leading to a mutated and non-cleavable form of HO site without chromosomal rearrangement and would maintain the *URA3* marker on chromosome VII. This would lead to the formation of Ura^+^ survivors. (type d, Figure 3). Altogether, the differential repair of both DSBs on chromosome VII chromatids gave rise to the observed Ura^+^/Ura^−^ phenotypes of survivor colonies.

### Absence of Pol32 impedes template switching and forces crossover formation

An important aspect that remains unclear is the apparent instability of the BIR fork, which may be cleaved during its advance, promoting template switching or producing half-crossovers. All essential DNA replication factors except those for pre-replication complex assembly are required for BIR [14], playing in favor of fork stability. However, the DNA damage checkpoint is activated during BIR and dNTP levels are elevated to facilitate repair, which is thought to happen in the G2 phase of the cell cycle [11], [13], [55]. Consequently, DNA synthesis during BIR has been found to be highly inaccurate [56] and replication fork progression may be perturbed by the absence of S phase-specific factors. Interestingly, the nonessential DNA polymerase δ subunit Pol32 seems to represent a key factor for BIR completion but performs a function that is still unknown. In our assay, we observed that *pol32Δ* mutants had a clear defect in producing translocations, but not as strong as the one observed in *rad52Δ* and *rad51Δ* mutants (Figure 4A), confirming that some translocations were produced via BIR/SSA, in which extensive DNA synthesis would not be required. However, Pol32 became essential for translocations in the absence of *HMR* cleavage in the control *HMR-inc* strains (Figure 4B). In these strains, we could not detect any BIR intermediate (Figure 4C), meaning that Pol32 is required for DNA synthesis of few kilobases and that the latter was necessary for template switching. Surprisingly, we observed in *pol32Δ* survivors an extremely high level of chromosome III circularization, even in Ura^+^ survivors that likely repaired the DSB on chromosome VII by GC or NHEJ (Figure 7 and Figure S10). This would mean that DSBs induced at *HMR* could not be repaired by HR with *MAT* **a** *-inc* sequences in *pol32Δ* cells. Indeed, preferential formation of crossovers between *MAT* and *HMR* would lead to the extrusion of genes essential for viability, whereas crossovers between *HMR* and *HML* would lead to the formation of stable circular chromosomes. Preferential processing of recombination intermediates into crossovers in *pol32Δ* mutants have been reported in other studies, in which BIR events were aborted and resulted in half-crossovers [22], [23].

### Mus81 and Yen1 redundantly promote BIR

We determined the role of SSEs in the generation of chromosomal rearrangements using our assay as the goal to identify the nucleases that are required during BIR. Overall, we observed a significant decrease of chromosomal rearrangements in *mus81Δ* single mutants that was aggravated in *mus81Δ yen1Δ* mutants (Figure 4A). We have confirmed that the frequency of translocants only decreased in the *mus81Δ yen1Δ* mutants in the *HMR-inc* background (Figure 4B), in which BIR, and not BIR/SSA, is expected to occur. This indicates that Mus81 may play a role in BIR/SSA and that Mus81 functions can be fully taken over by other proteins during BIR. However, our data show that both Mus81 and Yen1 carry out redundant or equivalent activities, which are needed for BIR completion. Mus81 and Yen1 have already been implicated in DSB repair by recombination but not directly in BIR. Mus81 has been shown to act at replication forks. It has been proposed that Mus81 could cleave stalled forks but also to participate in recombination-mediated repair of cleaved or collapsed forks to allow their restart in yeast and humans [32]–[34], [57]. Mus81 is also required in humans for telomere recombination to allow proliferation of telomerase-negative cancer cells [58]. Formally, both mechanisms of replication fork restart and telomere recombination are equivalent to BIR. Yen1 roles in recombination have been revealed in the absence of Mus81. While *yen1Δ* mutants are repair proficient, *mus81Δ yen1Δ* double mutants exhibit a higher sensitivity to DNA-damaging agents that disturb replication fork progression than *mus81Δ* mutants [28], [30], [31]. Together, these data point out that Mus81 and Yen1 may promote a replication fork restart mechanism. *In vitro*, Yen1 is a specialized Holliday junction resolvase [26] whereas the Mus81-Mms4 complex prefers branched DNA substrates that contain a discontinuity or a nick adjacent to the branch point, but also cleaves normal HJs [27], [59]–[61]. Here, we have demonstrated genetically that both Mus81 and Yen1 were required for efficient BIR. According to previously published data, we propose that Mus81 and Yen1 would act to establish the replication fork required for BIR by processing recombination intermediates such as D-loops or HJs. Nevertheless, BIR still occurred at a low frequency in *mus81Δ yen1Δ* mutants, suggesting that other factors could promote this critical step of BIR in the absence of Mus81 and Yen1.

### Interplay between Slx4, Mus81, and Yen1 causes template switching and half-crossovers

Our results are consistent with additional roles of Mus81 and Yen1 in later steps of BIR. We have demonstrated that Mus81, Slx4 and Yen1 were required together for efficient template switching during BIR. The *mus81Δ slx4Δ yen1Δ* mutants showed an increased occurrence of T7/3*-MAT* translocations (Figure 5B), which we infer as being partly due to a defect in template switching from *MAT* to the *HM* loci (Figure 6). However, we did not observe any increase of T7/3*-MAT* in *mus81Δ slx1Δ yen1Δ* mutants, even though Slx1 is the catalytic subunit of Slx1-Slx4 nuclease heterodimer. On the contrary, we observed a WT or decreased level of T7/3*-MAT* translocations in all *slx1Δ* mutants. We concluded that Slx4 and Slx1 act independently in BIR, presumably because of Slx4 additional functions apart from Slx1 at the replication fork [51], [62], [63]. Regarding the involvement of SSEs in half-crossover production, the absence of Mus81 or Slx1 significantly decreased the amount of circular chromosomes III among translocants (Figure 7C). Notably, no further decrease was observed when removing Mus81, Slx1/Slx4 or Yen1, all of which have been involved in crossover formation during meiosis in yeast [64], [65]. This could be due to the involvement of other nucleases such as Mlh3 and Exo1, as recently reported during the revision of this manuscript [65].

Our assay does not permit a direct analysis of the role of SSEs in half-crossover since the formation of circular chromosomes III is limited by the frequencies of translocations, template switching and *HMR* cleavage. In principle, *HML* could also be cleaved upon invasion so that an *HMR*/*HML* double cleavage could lead to a circular chromosome III by an SSA-like mechanism. However, this hypothesis is not supported by our results as we observed a similar frequency of circular chromosomes III in *rad1Δ* mutants and WT. Instead, circularization of chromosome III via SSA would generate a heterologous single-stranded DNA overhang that would require Rad1 for its removal [66] and, indeed, we have observed a requirement of Rad1 in the formation of T7/3-*HMR* translocations via BIR/SSA in our assay (Figure 5 and Figure 6).

Despite the limitations of our assay, our genetic data suggest an interesting interaction between Mus81, Yen1 and Slx4 SSEs. Whereas *mus81Δ* translocants showed a decrease in the frequency of circular chromosomes III, *mus81Δ slx4Δ* translocants showed a significant increase, which was suppressed in the additional absence of Yen1 (Figure 7C). These results suggest that Slx4 may have a specific role in regulating the ability of Mus81 and Yen1 to catalyze half-crossovers. It has been previously shown that Mus81 is involved in half-crossovers following BIR [23] and that Mus81 and Yen1 independently promote crossovers during gene conversion, Yen1 serving as a backup function in *mus81Δ* cells [30]. However, here we uncover two parallel pathways, one using Mus81 and Slx4 and the other Yen1. This is in agreement with a similar involvement recently described for these nucleases in two pathways of crossover formation during sister-chromatid recombination [67]. It remains unclear how Slx4 may regulate Mus81 and Yen1. A recent cell-cycle analysis of Mus81-Mms4 and Yen1 revealed that their catalytic activities are regulated by phosphorylation events. In mitotic cells, Mus81-Mms4 is hyperactivated by Cdc5-mediated phosphorylation at G2/M while Yen1 is activated later by dephosphorylation in M phase [27]. Nevertheless, it remains unknown if Yen1 can be activated earlier in the absence of Mus81 or upon DSB induction. Mec1/Tel1 kinases phosphorylate Slx4 in response to DNA damage [50], [51] and may participate in modulating context-specific protein interactions between Slx4, Mus81 and Yen1 and allow substrate accessibility to activated Mus81 and Yen1. Altogether, our results suggest that Slx4 plays a central role during BIR. Slx4 may regulate Mus81 and Yen1, whose cleavage activities are required for replication fork establishment and could either cause template switching or half-crossovers.

In the case of one-ended DSBs, it has been proposed that dynamic displacement of the invading strand out of the D-loop would contribute to template switching [16]. This implies that the invading strand would be displaced early during BIR, after a short tract of DNA synthesis. Nevertheless, events of template switching have been observed in later steps of BIR, as far as 10-kb downstream of the site of invasion [16], [23]. Such a synthesis would expose long tracts of single-stranded DNA if it were the result of the sole extension of the invading strand. Despite the fact that such long single-stranded DNA tails have been involved in gene conversion events monitored in mitotic gap repair assays, we propose that, at some point, priming of lagging strand synthesis would ensure a better protection of the recombination intermediates, safeguarding genome stability. Thus, we propose that template switching events would happen after the establishment of the BIR fork and priming of lagging strand synthesis. *In vitro* data showed that canonical replication forks are among the preferred substrates of Mus81-Mms4 and Yen1 [25], [68], therefore we propose that Mus81, Slx4 and Yen1 would act on the replication fork during BIR to cause template switching and half-crossovers.

### An integrated model to explain BIR–mediated chromosomal rearrangements and the role of structure-selective endonucleases

**Figure 8:**
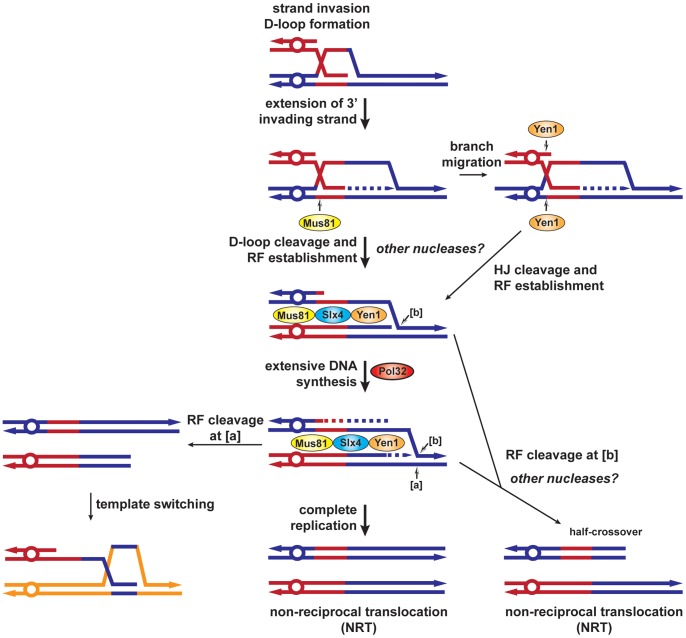
BIR model showing SSEs involvement in template switching and half-crossovers. After invasion of the homologous template by one end of the DSB, a D-loop is formed and extended by the priming of DNA synthesis from the invading strand 3′ end. The D-loop can be specifically cleaved by Mus81 or branch migrated to create a HJ cleavable by Yen1, to establish a replication fork. Extensive replication, which requires Pol32, would complete BIR and generate a non-reciprocal translocation (NRT). Alternatively, [a] cleavage of the replication fork by Mus81-Slx4-Yen1 would allow re-invasion of the same template or template switching. [b] cleavage of the replication fork by Mus81-Slx4-Yen1 would terminate the BIR event, causing a half-crossover (NRT). RF, replication fork, HJ, Holliday junction.

Our results together with previous data permit us to propose a new model for BIR and the role of the different SSEs used in this study (Figure 8). During HR, priming of synthesis from the 3′ invading end extends the initial D-loop and failure to capture the other DSB end would promote BIR. We propose that Mus81 would cleave the extended D-loop structure to allow the establishment of a replication fork. In the absence of Mus81, branch migration of the D-loop would create an intact Holliday junction, which could be processed by Yen1 with the same outcome. Pol32 would promote extensive DNA synthesis and complete replication would generate a non-reciprocal translocation (NRT). We propose that the BIR fork could stall and be processed by Mus81-Slx4-Yen1 to cause template switching ([a], Figure 8). Differential cleavage of the BIR fork by Mus81-Slx4-Yen1 would terminate BIR at the expense of a half-crossover ([b], Figure 8).

Altogether, this work brings a clearer view about the involvement of SSEs in the BIR mechanism of DSB repair. Importantly, we show that SSEs are involved in replication template switching and half-crossovers, which generate complex chromosomal rearrangements and prolonged cycles of genomic instability. Such events are thought to be at the origin of various genomic disorders and cancer development [24], [69].

## Materials and Methods

### Yeast strains and plasmids

All *Saccharomyces cerevisiae* yeast strains used in this study are in W303-1a background (*his3-11*, *15 leu2-3*, *112*, *trp1-1 ura3-1 ade2-1 can1-100 rad5-535*) [70] and harbor *MAT* **a** *-inc*, *ade3Δ::gal-HO* and *leu2Δ::SFA1* alleles [53]. The *MAT* **a** *-inc HMR-inc* strain was obtained by mating-type switching inducing HO expression in a *MAT* ***α*** *HMR-inc* strain. Independent survivors to HO expression were selected and the *MAT* and *HMR* loci were sequenced to verify the *MAT* **a** *-inc HMR-inc* genotype. Deletion mutants were either obtained by the PCR-based gene replacement method (verified by PCR and Southern) or by genetic crosses (verified by tetrad analysis). Deletion of *MAT* is only partial (*matΔYZ*) because of the presence of other genes overlapping with *MAT*. Only the Y and Z sequences, containing the HO cut site, have been removed. Insertion of a HO-cleavable 240-bp *HMR* fragment at the *ADH4* locus has been conducted as follows. Two 5′ and 3′*ADH4* fragments were amplified by PCR with the following primer pairs ADH4-5′#1 GCGCGCGGTACCGAATTCAAACCGCTGATTACATCAAA and ADH4-5′#2 GCGCGCAGATCTATCGATCTCGAGTCTAGACTAGACCAGTAGCAGCAGTC, and ADH4-3′#1 GCGCGCAGATCTGCTAGCACTAGTGGATCCCTTAGTCGCTGCATACAAAG and ADH4-3′#2 GCGCGCGAGCTCGAATTCGCACACGCATAATTGACGTT. These two fragments were cloned by the gap repair method in pBluescript II(SK+) previously digested by *Kpn*I and *Sac*I to create pBP99 plasmid. A *Bgl*II-*Bam*HI *URA3* containing fragment from sp392 plasmid [71] was then cloned in pBP99 digested with *Bgl*II to create pBP102. Finally, the *HMR* fragment was amplified from genomic DNA with the primer pair HO-HMR-Hind3 GCGCGCAAGCTTCAACCACTCTACAAAACCAAAACCA and HO-HMR-Nhe1 GCGCGCGCTAGCAGAAGAAGTTGCAAAGAAATGTGGC and cloned into pBP102 after digestion with *Hind*III and *Nhe*I, to create pBP102-HO. pBP102-HO was linearized with *Pvu*II and transformed into yeast. Integration was selected by uracile prototrophy and verified by PCR and Southern analysis.

### Determination of survival and translocation frequencies

Yeast cells were grown in yeast extract-peptone-adenine-dextrose (YPAD) until reaching the exponential phase of growth, appropriately diluted with H_2_O and plated on synthetic complete (SC) medium containing either 2% glucose or 2% galactose as a carbon source. Survivor colonies on galactose-containing plates were then restreaked on YPAD plates and replica-plated on SC plates containing 5-FOA (USBiological), a drug that generates a toxic metabolite in Ura^+^ cells, or on SC plates lacking uracil. Frequencies were calculated as follows: survival frequency = cfu galactose/cfu glucose; translocants frequency = (cfu galactose Ura−5-FOAr+(cfu galactose Ura+5-FOAr)/2)/cfu glucose. 96 to 288 survivor colonies, recovered from 2 to 3 independent induction experiments, were analyzed for each strain tested. Statistical analysis was performed using the χ^2^ test with Yates' correction.

### Kinetic analysis of BIR intermediates by PCR and Southern analysis

Yeast cells were grown at 30°C in liquid YPAD until reaching the exponential phase of growth, washed twice with synthetic complete medium SGL (3% glycerol, 2% lactate) and cultured overnight in SGL until reaching an OD_600 nm_≈0.5 when galactose was added at a final concentration of 2%. Cells were taken at different times after galactose induction and genomic DNA was extracted in agarose plugs according to standard procedures. Agarose plugs were incubated twice in 200 µl 1× β-Agarase I reaction buffer for 30 min, melted at 65°C for 10 min, equilibrated at 42°C for 15 min and treated with β-Agarase I (New England BioLabs) at 42°C for 1 h before PCR amplification. These were performed with 250 ng of genomic DNA (estimated with NanoDrop, Thermo Scientific) in a total volume of 30 µl in the following conditions: 1× Phusion HF buffer, 200 µM each dNTP, 0.6 U Phusion DNA polymerase (Finnzymes), 0.5 µM each primer. Samples were denatured for 45 s at 98°C, then cycled 25–35 times with 20 s denaturation (98°C), 30 s annealing (57°C) and 45 s extension (72°C) followed by a final extension step of 5 min at 72°C. PCR was performed with primer p7 GCACACGCATAATTGACGTT and primers p3-M GAAGACTTGTGGCGAAGA, p3-R CCAACATTTAGGAAAAAACG or p3-L CGGATGGCACAAGGAACACGCATTT. Control PCR was performed with primers corresponding to *ACT1* locus, ACT1up TTCACGCTTACTGCTTTTTTC and ACT1low CAAGGCGACGTAACATAGTTT. PCR products were subjected to gel electrophoresis in 0.8% agarose and stained with ethidium bromide. Instead of β-Agarase I treatment, plugs were digested with 30 U of *Eco*RV restriction enzyme for 5 h at 37°C and loaded in a 1% agarose gel for Southern analysis. Electrophoresis was run at 80 V for 16 h30 and DNA was transferred into Hybond-XL membranes (GE Healthcare) in alkaline conditions. Membranes were probed with dCT^32^P-labelled PCR fragments obtained with ADH4-3′#1 and ADH4-3′#1 primers (7L probe). Quantification of DNA signals was made relative to the total DNA of each lane and was performed using ImageGauge 4.2 (Fujifilm) program.

### Pulsed-field gel electrophoresis (PFGE) analysis

For each strain, 28 to 84 independent Ura- 5-FOAr survivor colonies were grown in 2,5 ml of YPAD medium overnight at 30°C. Agarose plugs containing chromosomal DNA were made according to the manufacturer's instructions (Bio-Rad). *Asi*SI digestion was performed incubating agarose plugs twice in 1 ml 1× NEBuffer 4 for 30 min and digested in 200 µl 1× NEBuffer 4 with 30 U of *Asi*SI restriction enzyme for 5 h (New England Biolabs). Agarose gels (0.9%) were run in a Bio-Rad CHEF MapperXA apparatus for 16 h at 6 V/cm with a switch time of 70 s and for an additional 12 h at 6 V/cm with a switch time of 120 s. Then, gels were stained with ethidium bromide and DNA was transferred into Hybond-XL membranes (GE Healthcare) in alkaline conditions. Membranes were probed with dCT^32^P-labelled PCR fragments obtained with primers ADH7#1 TGTTGGCTAAAGCTATGG and ADH7#2 TTCTTCGCTGATCGG (3R1 probe), ARS315#1 AAACCAGTCTTTAACCGCCATAATG and ARS315#2 CAGAGCCCAAGAGATAGCCGAACTT (3R2 probe), and with primers HML+HMR-F CAAACATCTTAGTAGTGTCTGAGGA and HML+HMR-R CTGTAATTTACCTAAGTTACCAGAG (X probe). Chromosomal rearrangements different from T7/3*-MAT* or T7/3*-HMR* translocations or circular chromosomes III and revealed by the PFGE analysis were not included in the statistical analyses.

## Supporting Information

Figure S1 Survival frequency to DSB induction in the WT and SSE mutants. Graphical plotting of survival frequency of each strain tested. See Material and Methods for more details. †, frequency<10^−4^. Error bars represent standard deviations.
(TIF)

Figure S2 PFGE analysis of translocations in mixed Ura^+^ 5-FOA-resistant colonies. Translocations between chromosomes VII and III were detected by PFGE followed by Southern analysis using the 7L (A) and the 3R1 (B) probe. Chromosomal DNA samples were extracted from previously separated Ura^+^ and 5-FOA-resistant cells. Black arrows indicate the positions of the T7/3-*HMR* translocations and of the linear chromosome III (Chr. III).
(TIF)

Figure S3 DNA invasion at *HMR* causes its cleavage. (A) Schematic representation of chromosomes VII and III in our assay. Localization of the 7L and X probes is indicated. (B) Detection of HO cleavage on chromosome VII, as monitored by Southern analysis using the 7L probe in the WT and *hmlΔ matΔYZ* strains. DNA samples were extracted at different intervals after HO induction with galactose. Positions of the bands corresponding to the uncut and cut chromosome VII (chr. VII) are indicated. (C) (D) Detection of *HMR* cleavage product, as monitored by Southern analysis using the X probe in the WT and *hmlΔ matΔYZ* strains and the *hmlΔ matΔYZ* strain without HO cut site in chromosome VII. DNA samples were extracted at different intervals after HO induction with galactose. Quantification of cleavage at each time point is indicated at the bottom. Positions of the bands corresponding to *MAT*, *HMR*, *HML*, cut *MAT*, cut *HMR*, cut *HML* and *matΔYZ* are indicated. GAL, galactose; h, hours.
(TIF)

Figure S4 Analysis of repair synthesis products in WT cells, as monitored by PCR. Experiments were performed as in Figure 2B except that in this case only 25 cycles of PCR amplifications were done.
(TIF)

Figure S5 Genetic analysis of T7/3 translocations in SSE mutants. PFGE followed by Southern analysis using the 3R1 probe of Ura^−^ 5-FOAr survivors in the WT and different mutants combination strains. Black arrows indicate the positions of the T7/3-*MAT* and T7/3-*HMR* translocations and of the linear chromosome III (Chr. III).
(TIF)

Figure S6 Genetic analysis of T7/3 translocations in SSE mutants. Details as in Figure S5.
(TIF)

Figure S7 PFGE analysis of translocations in the *HMR-inc* WT strain. (A) Schematic representation of chromosomes III. Localization of the 3L and 3R1 probes is indicated. PFGE followed by Southern analysis using the 3R1 (B) or 3L probes (C) of Ura^−^ 5-FOAr survivors in the control *HMR-inc* WT strain. Black arrows indicate the positions of the T7/3-*MAT* and T7/3-*HMR* translocations and the circular and linear chromosomes III (Chr. III).
(TIF)

Figure S8 Genetic analysis of chromosome III circularization in SSE mutants. PFGE followed by Southern analysis using the 3R2 probe of Ura^−^ 5-FOAr survivors in the WT and different mutants combination strains. Black arrows indicate the positions of T7/3-*MAT* translocations, and the circular and linear chromosomes III (Chr. III). A red asterisk (*) indicates residual labeling of the 3R1 probe.
(TIF)

Figure S9 Genetic analysis of chromosome III circularization in SSE mutants. Details as in Figure S8.
(TIF)

Figure S10 Analysis of chromosome III circularization in Ura^+^ survivors. (A), (B) Southerns from Figure 1D and Figure S2 were re-hybridized with the 3R2 probe to show the presence of circular chromosomes III in the wells. (C), (D) PFGE followed by Southern analysis using the 3R1 and 3R2 probes of *pol32Δ* Ura^+^ 5-FOAs survivors. (E) Frequency of Ura^+^ survivors carrying a circular chromosome III in WT and *pol32Δ* strains. **, differences with the WT statistically significant (p<0.01, χ^2^ with Yates' correction).
(TIF)

Table S1 Statistical analysis of PFGE data. The number of translocants analyzed by PFGE and those that contained a T7/3-*MAT* translocation or a circular chromosome III are indicated for each genotype. Numbers in parentheses correspond to the percentage of the total. The differences between the WT were analyzed using a χ^2^ test with Yates' correction. *, statistically significant P values are 0.05 for χ^2^>3.84 and 0.01 for χ^2^>6.63. n.d., not determined.
(DOC)
