# Supplementary figures and images for: Complex Chromosomal Rearrangements Mediated by Break-Induced Replication Involve Structure-Selective Endonucleases
Source: PLoS Genet. 2012 Sep 27;8(9):e1002979. doi: 10.1371/journal.pgen.1002979 (PMC3459980; doi:10.1371/journal.pgen.1002979)

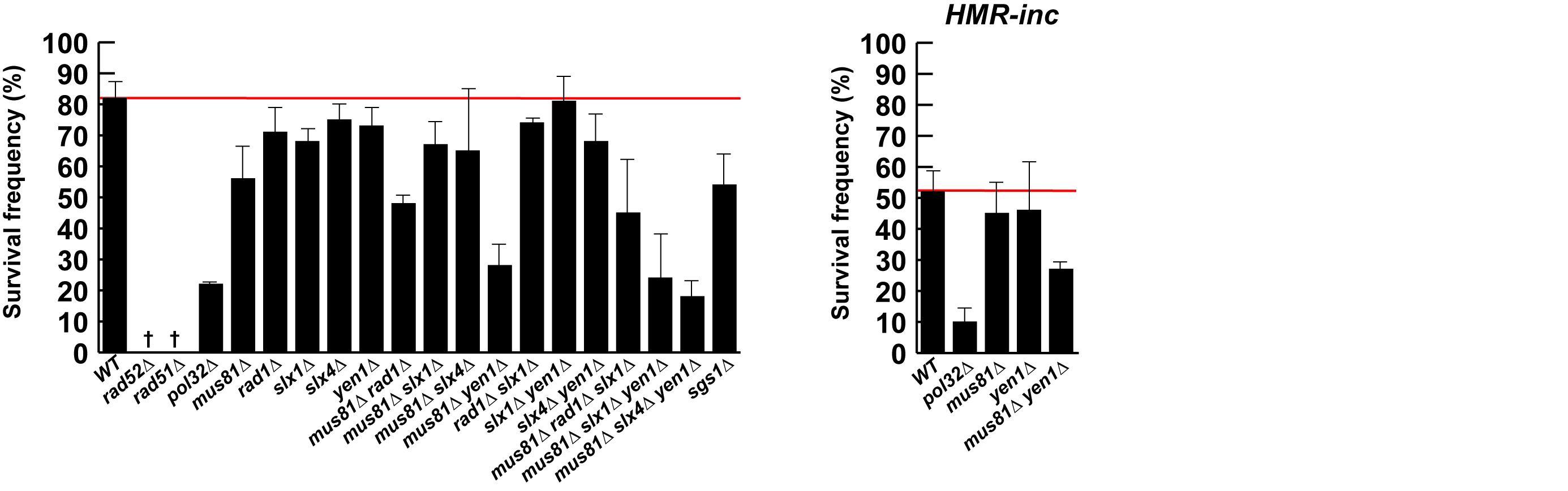

Supplement: Figure S1 — Survival frequency to DSB induction in the WT and SSE mutants. Graphical plotting of survival frequency of each strain tested. See Material and Methods for more details. †, frequency<10−4. Error bars represent standard deviations. (TIF) [file pgen.1002979.s001.tif]

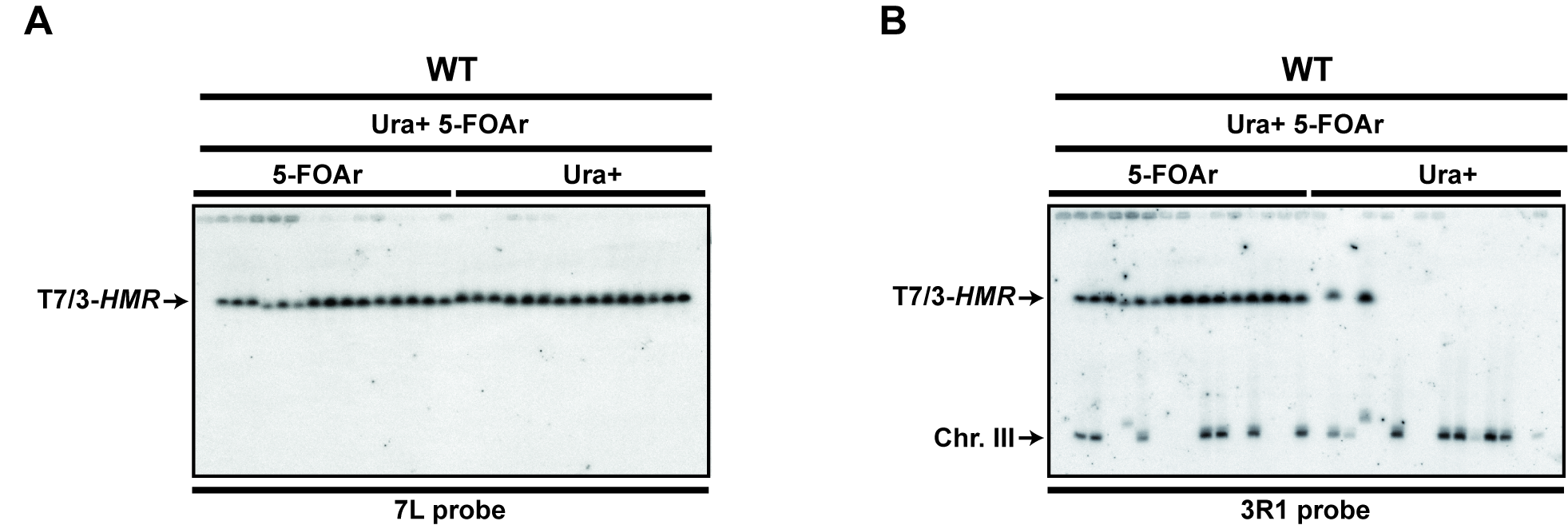

Supplement: Figure S2 — PFGE analysis of translocations in mixed Ura+ 5-FOA-resistant colonies. Translocations between chromosomes VII and III were detected by PFGE followed by Southern analysis using the 7L (A) and the 3R1 (B) probe. Chromosomal DNA samples were extracted from previously separated Ura+ and 5-FOA-resistant cells. Black arrows indicate the positions of the T7/3-HMR translocations and of the linear chromosome III (Chr. III). (TIF) [file pgen.1002979.s002.tif]

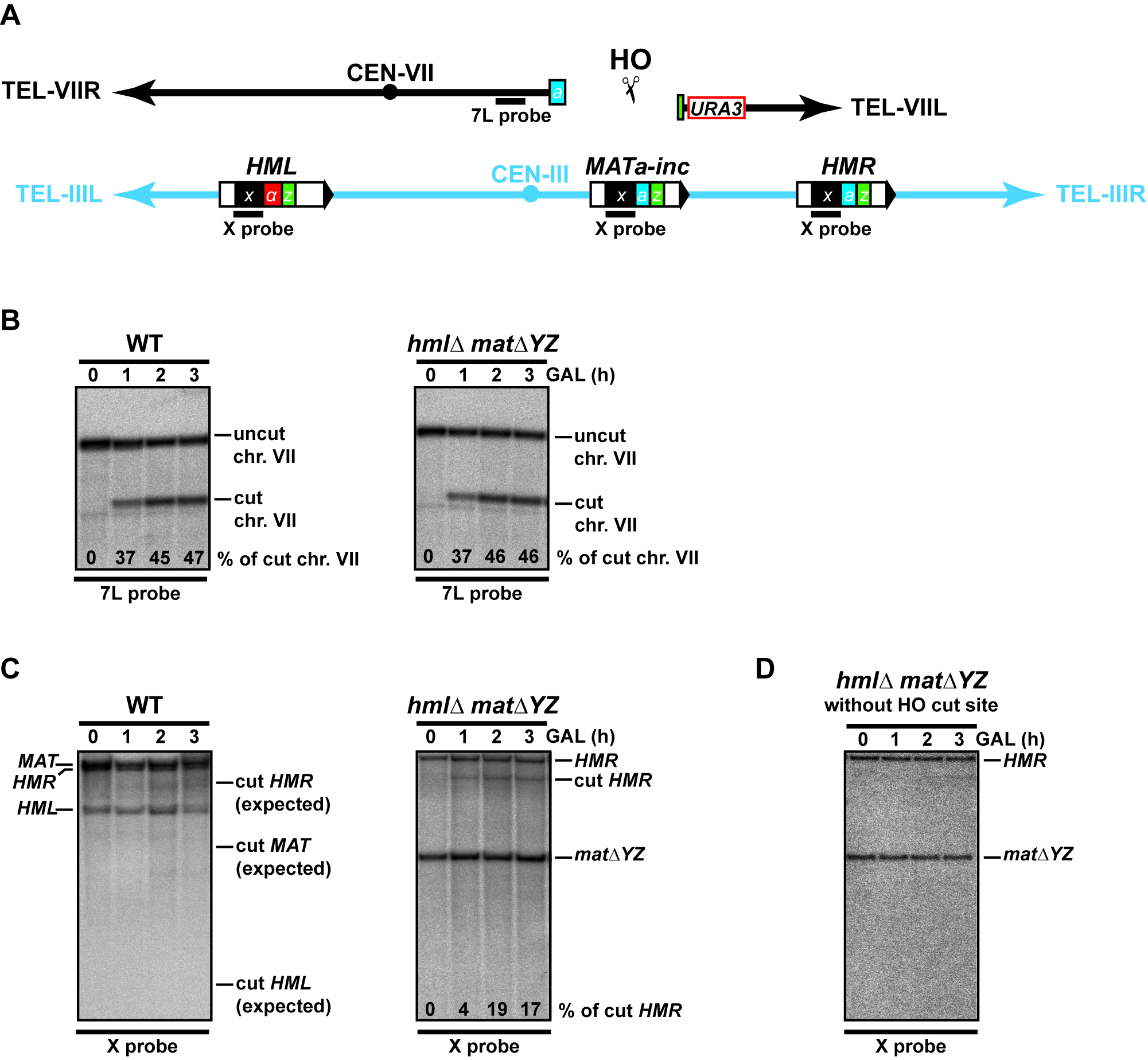

Supplement: Figure S3 — DNA invasion at HMR causes its cleavage. (A) Schematic representation of chromosomes VII and III in our assay. Localization of the 7L and X probes is indicated. (B) Detection of HO cleavage on chromosome VII, as monitored by Southern analysis using the 7L probe in the WT and hmlΔ matΔYZ strains. DNA samples were extracted at different intervals after HO induction with galactose. Positions of the bands corresponding to the uncut and cut chromosome VII (chr. VII) are indicated. (C) (D) Detection of HMR cleavage product, as monitored by Southern analysis using the X probe in the WT and hmlΔ matΔYZ strains and the hmlΔ matΔYZ strain without HO cut site in chromosome VII. DNA samples were extracted at different intervals after HO induction with galactose. Quantification of cleavage at each time point is indicated at the bottom. Positions of the bands corresponding to MAT, HMR, HML, cut MAT, cut HMR, cut HML and matΔYZ are indicated. GAL, galactose; h, hours. (TIF) [file pgen.1002979.s003.tif]

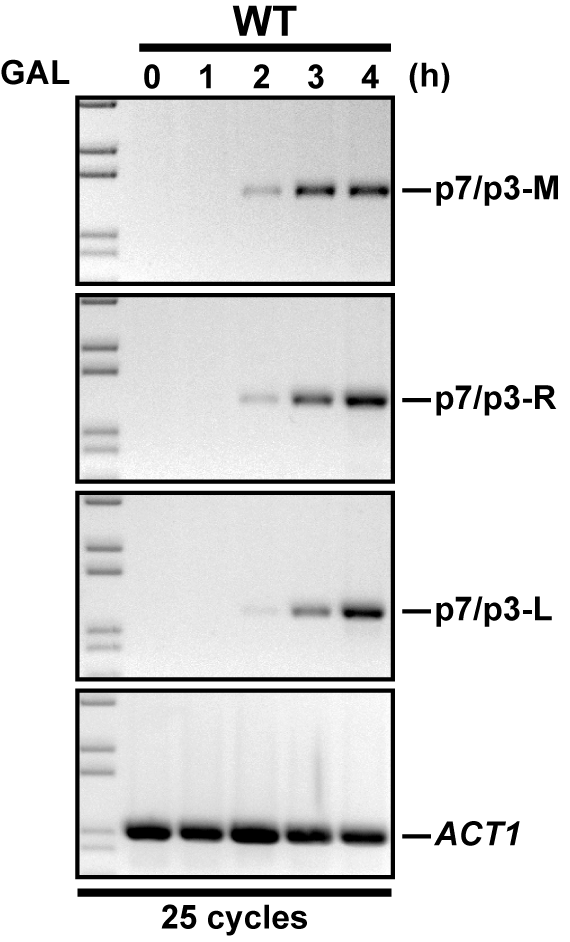

Supplement: Figure S4 — Analysis of repair synthesis products in WT cells, as monitored by PCR. Experiments were performed as in Figure 2B except that in this case only 25 cycles of PCR amplifications were done. (TIF) [file pgen.1002979.s004.tif]

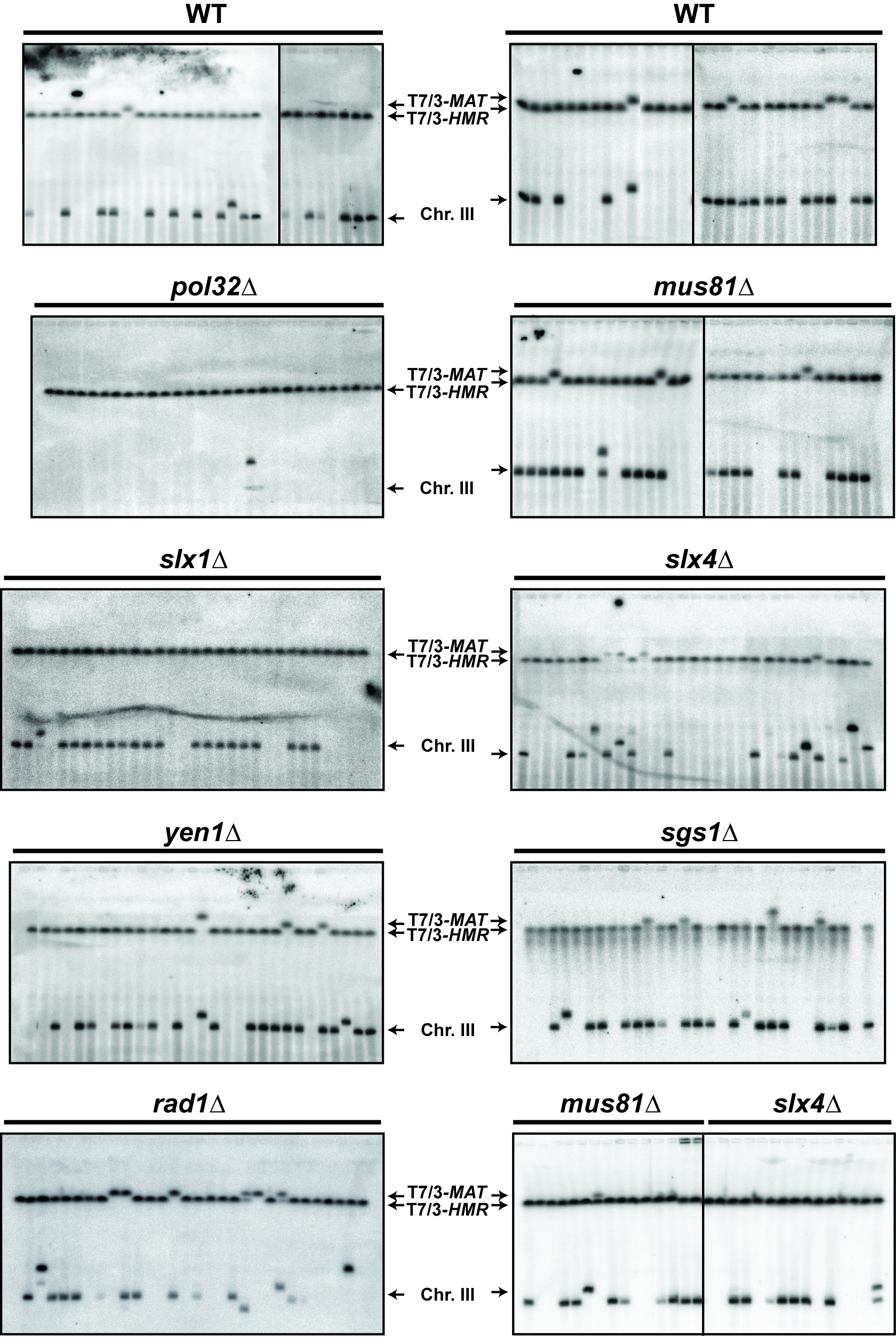

Supplement: Figure S5 — Genetic analysis of T7/3 translocations in SSE mutants. PFGE followed by Southern analysis using the 3R1 probe of Ura− 5-FOAr survivors in the WT and different mutants combination strains. Black arrows indicate the positions of the T7/3-MAT and T7/3-HMR translocations and of the linear chromosome III (Chr. III). (TIF) [file pgen.1002979.s005.tif]

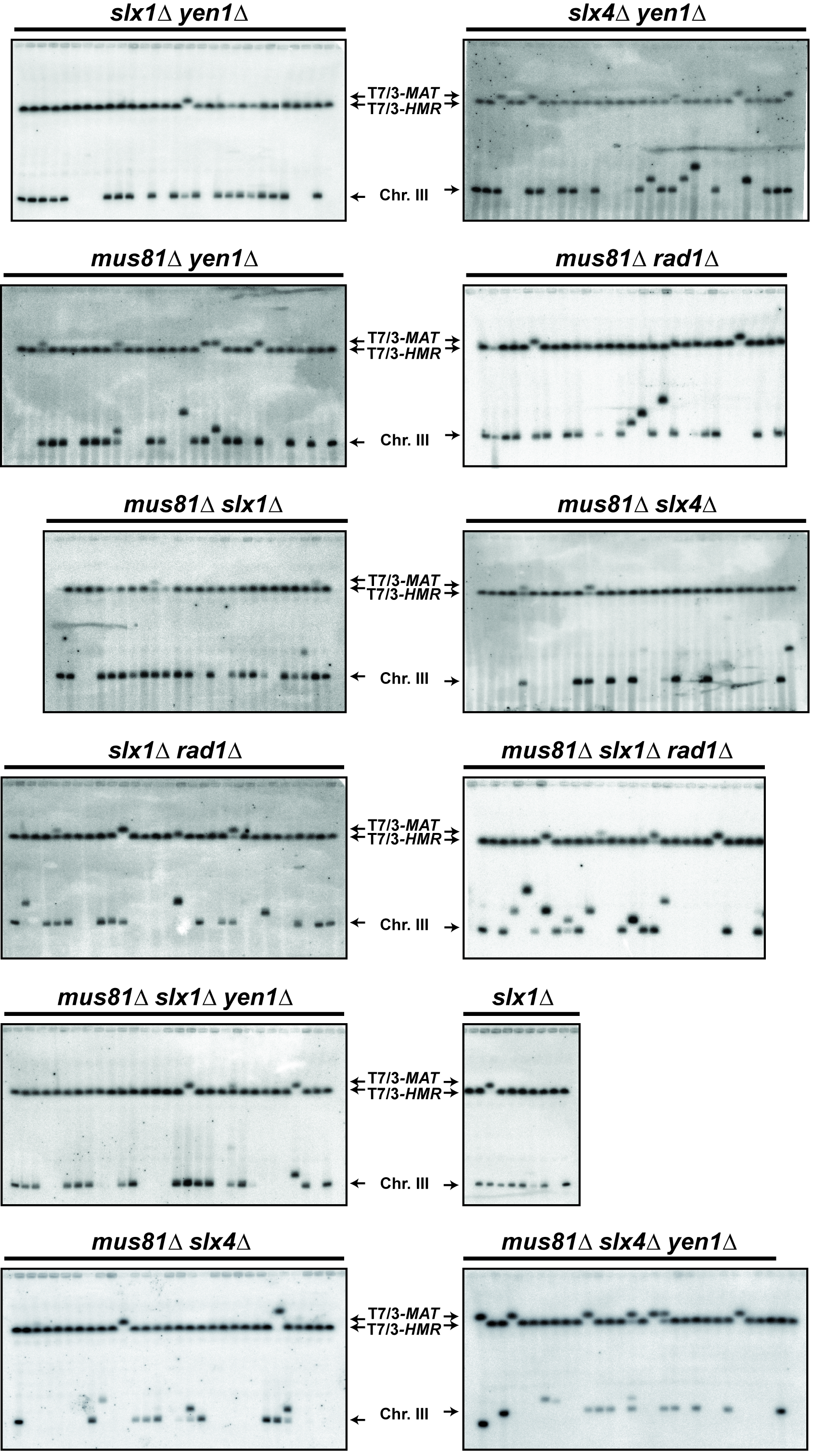

Supplement: Figure S6 — Genetic analysis of T7/3 translocations in SSE mutants. Details as in Figure S5. (TIF) [file pgen.1002979.s006.tif]

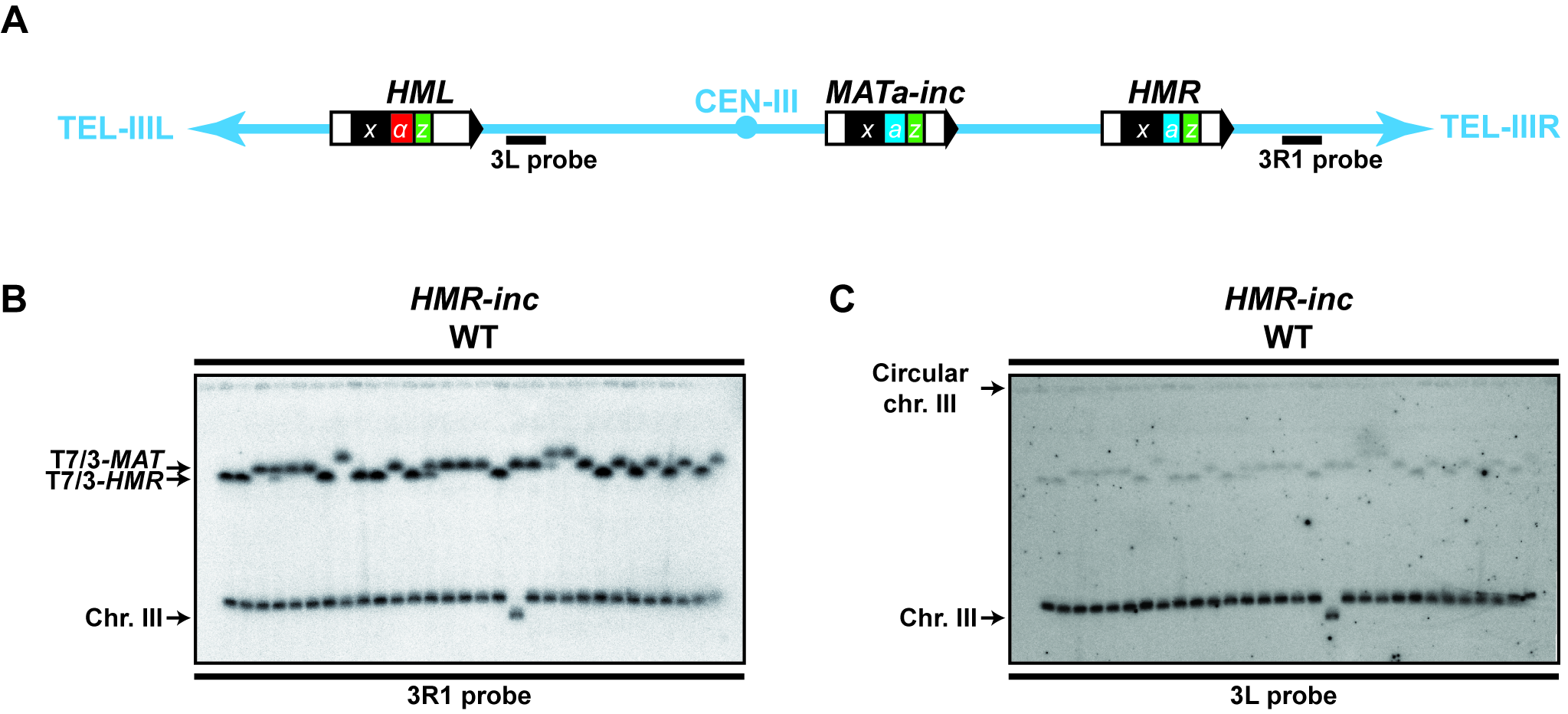

Supplement: Figure S7 — PFGE analysis of translocations in the HMR-inc WT strain. (A) Schematic representation of chromosomes III. Localization of the 3L and 3R1 probes is indicated. PFGE followed by Southern analysis using the 3R1 (B) or 3L probes (C) of Ura− 5-FOAr survivors in the control HMR-inc WT strain. Black arrows indicate the positions of the T7/3-MAT and T7/3-HMR translocations and the circular and linear chromosomes III (Chr. III). (TIF) [file pgen.1002979.s007.tif]

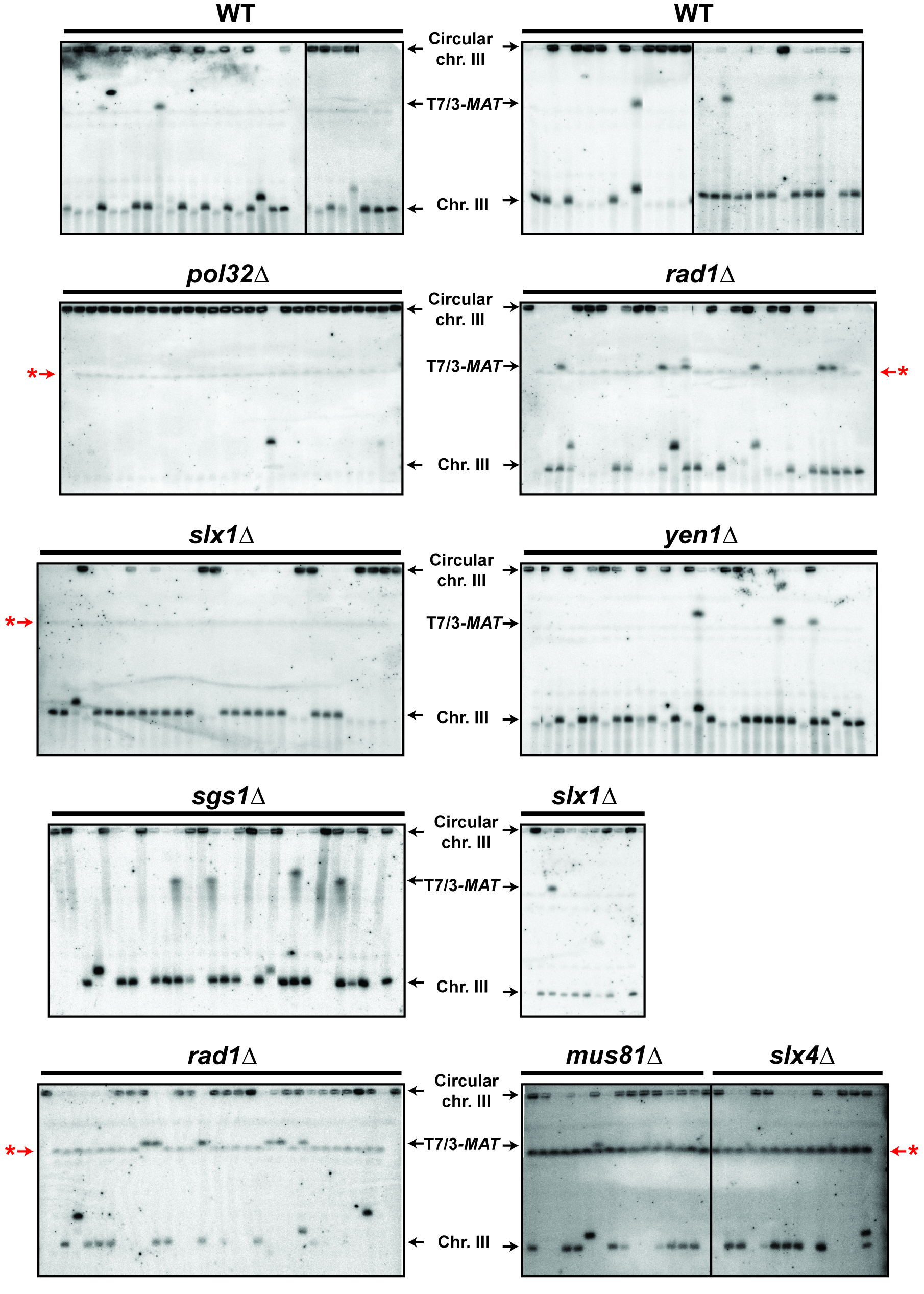

Supplement: Figure S8 — Genetic analysis of chromosome III circularization in SSE mutants. PFGE followed by Southern analysis using the 3R2 probe of Ura− 5-FOAr survivors in the WT and different mutants combination strains. Black arrows indicate the positions of T7/3-MAT translocations, and the circular and linear chromosomes III (Chr. III). A red asterisk (*) indicates residual labeling of the 3R1 probe. (TIF) [file pgen.1002979.s008.tif]

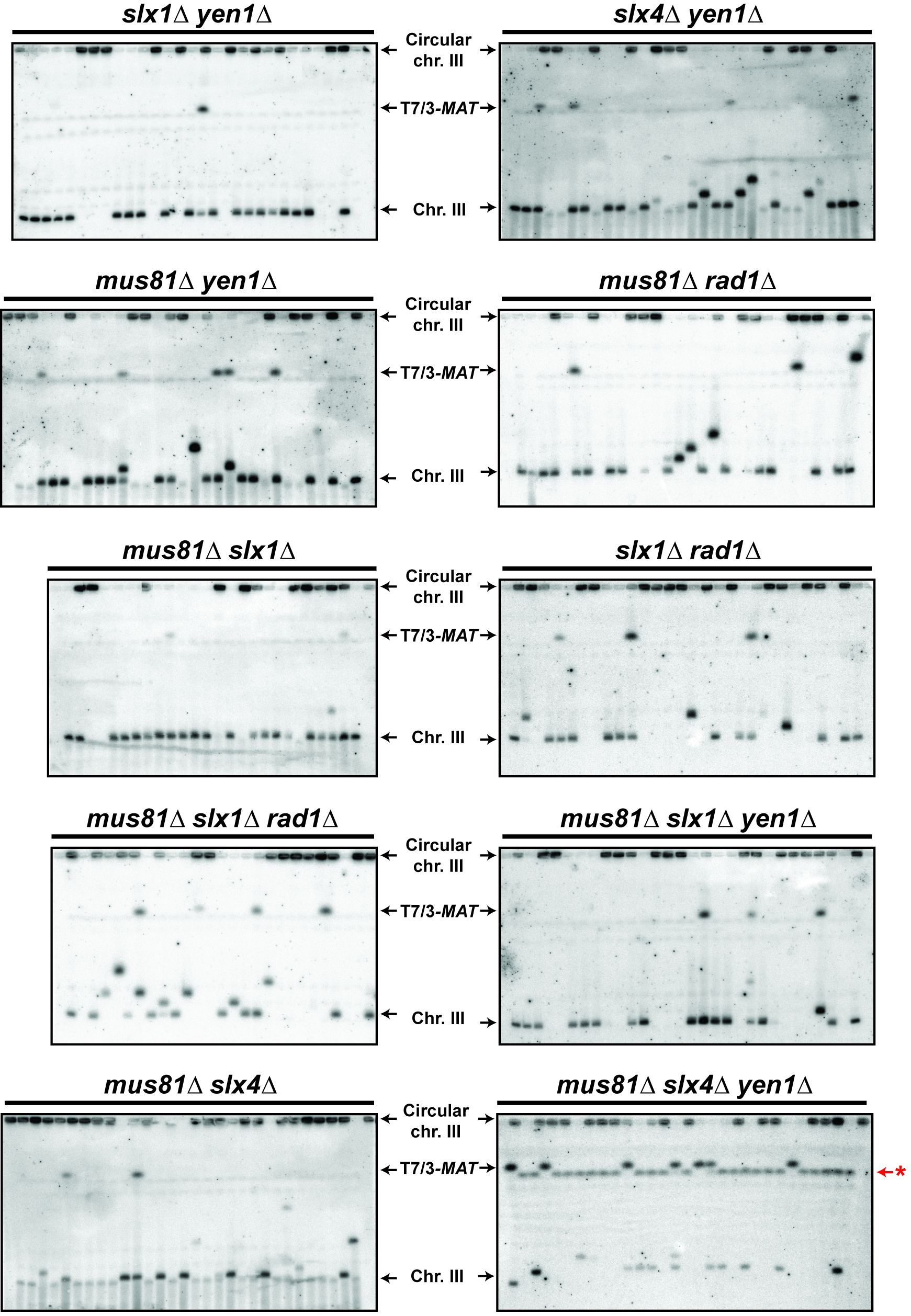

Supplement: Figure S9 — Genetic analysis of chromosome III circularization in SSE mutants. Details as in Figure S8. (TIF) [file pgen.1002979.s009.tif]

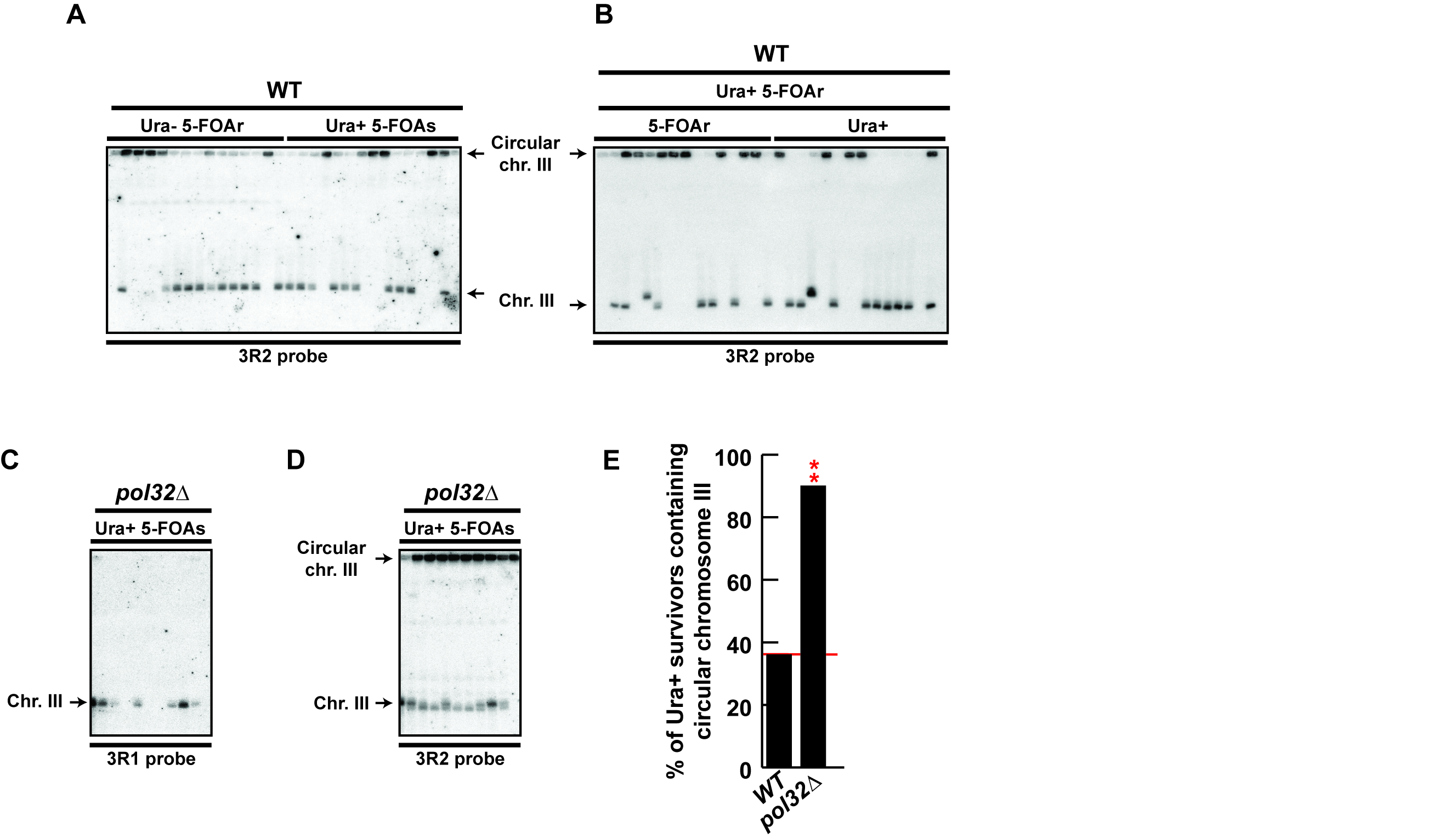

Supplement: Figure S10 — Analysis of chromosome III circularization in Ura+ survivors. (A), (B) Southerns from Figure 1D and Figure S2 were re-hybridized with the 3R2 probe to show the presence of circular chromosomes III in the wells. (C), (D) PFGE followed by Southern analysis using the 3R1 and 3R2 probes of pol32Δ Ura+ 5-FOAs survivors. (E) Frequency of Ura+ survivors carrying a circular chromosome III in WT and pol32Δ strains. **, differences with the WT statistically significant (p<0.01, χ2 with Yates' correction). (TIF) [file pgen.1002979.s010.tif]
