# Supplementary material for: Complex Chromosomal Rearrangements Mediated by Break-Induced Replication Involve Structure-Selective Endonucleases
Source: PLoS Genet. 2012 Sep 27;8(9):e1002979. doi: 10.1371/journal.pgen.1002979 (PMC3459980; doi:10.1371/journal.pgen.1002979)
Supplement: Table S1 — Statistical analysis of PFGE data. The number of translocants analyzed by PFGE and those that contained a T7/3-MAT translocation or a circular chromosome III are indicated for each genotype. Numbers in parentheses correspond to the percentage of the total. The differences between the WT were analyzed using a χ2 test with Yates' correction. *, statistically significant P values are 0.05 for χ2>3.84 and 0.01 for χ2>6.63. n.d., not determined. (DOC) [file pgen.1002979.s011.doc]

| genotype | # of translocants analyzed by PFGE | # of translocants with T7/3-MAT translocation | χ^2^ with Yate's correction | # of translocants with circular chromosome III | χ^2^ with Yate's correction |
| --- | --- | --- | --- | --- | --- |
| WT | 84 | 7 (8%) |  | 42 (50%) |  |
| *rad52∆* | 0 | n.d. | n.d. | n.d. | n.d. |
| *rad51∆* | 0 | n.d. | n.d. | n.d. | n.d. |
| *pol32∆* | 59 | 0 (0%) | 4.33* | 52 (88%) | 32.81* |
| *mus81∆* | 45 | 3 (7%) | 0.02 | 15 (33%) | 4.36* |
| *rad1∆* | 59 | 12 (20%) | 9.62* | 31 (53%) | 0.07 |
| *slx1∆* | 40 | 1 (3%) | 1.10 | 12 (30%) | 5.63* |
| *slx4∆* | 44 | 4 (10%) | 0.01 | 26 (59%) | 1.11 |
| *yen1∆* | 28 | 2 (7%) | 0.01 | 11 (39%) | 0.89 |
| *mus81∆ rad1∆* | 28 | 2 (7%) | 0.01 | 11 (39%) | 0.89 |
| *mus81∆ slx1∆* | 26 | 1 (4%) | 0.22 | 9 (35%) | 1.88 |
| *mus81∆ slx4∆* | 59 | 2 (3%) | 1.30 | 43 (72%) | 11.46* |
| *mus81∆ yen1∆* | 30 | 4 (13%) | 0.44 | 13 (43%) | 0.30 |
| *rad1∆ slx1∆* | 30 | 2 (8%) | 0.00 | 16 (53%) | 0.03 |
| *slx1∆ yen1∆* | 29 | 1 (3%) | 0.38 | 12 (41%) | 0.55 |
| *slx4∆ yen1∆* | 28 | 4 (14%) | 0.64 | 11 (39%) | 0.89 |
| *mus81∆ rad1∆ slx1∆* | 28 | 3 (11%) | 0.01 | 15 (54%) | 0.04 |
| *mus81∆ slx1∆ yen1∆* | 50 | 4 (8%) | 0.03 | 19 (38%) | 2.42 |
| *mus81∆ slx4∆ yen1∆* | 58 | 15 (26%) | 18.97* | 30 (58%) | 0.02 |
| *sgs1∆* | 28 | 3 (11%) | 0.01 | 13 (46%) | 0.04 |
